# Multifunctional Protein Hybrid Nanoplatform for Synergetic Photodynamic‐Chemotherapy of Malignant Carcinoma by Homologous Targeting Combined with Oxygen Transport

**DOI:** 10.1002/advs.202203742

**Published:** 2022-12-21

**Authors:** Song‐Yu Wu, Ya‐Xi Ye, Qing Zhang, Qian‐Jin Kang, Zhu‐Min Xu, Shen‐Zhen Ren, Fan Lin, Yong‐Tao Duan, Hao‐Jun Xu, Zi‐Yi Hu, Sui‐Sui Yang, Hai‐Liang Zhu, Mei‐Juan Zou, Zhong‐Chang Wang

**Affiliations:** ^1^ State Key Laboratory of Pharmaceutical Biotechnology School of Life Sciences Institute of Artificial Intelligence Biomedicine Engineering Research Center of Protein and Peptide Medicine Nanjing University Nanjing 210023 P. R. China; ^2^ Department of Pharmacology Department of Cell Biology School of Basic Medical Sciences Nanjing Medical University 101 Longmian Avenue, Nanjing Jiangning 211166 China; ^3^ Institute of Pharmaceutical Biotechnology School of Biology and Food Engineering Suzhou University Suzhou 234000 P. R. China; ^4^ Key Laboratory of Molecular Biophysics Hebei Province Institute of Biophysics School of Sciences Hebei University of Technology Tianjin 300401 China; ^5^ Henan provincial key laboratory of children's genetics and metabolic diseases Children's Hospital Affiliated to Zhengzhou University Zhengzhou University Zhengzhou 450018 China

**Keywords:** blood‐brain barrier, homologous targeting, multifunctional nanoplatform, protein hybrid, tumor hypoxic microenvironment

## Abstract

Photodynamic therapy (PDT) under hypoxic conditions and drug resistance in chemotherapy are perplexing problems in anti‐tumor treatment. In addition, central nervous system neoplasm‐targeted nanoplatforms are urgently required. To address these issues, a new multi‐functional protein hybrid nanoplatform is designed, consisting of transferrin (TFR) as the multicategory solid tumor recognizer and hemoglobin for oxygen supply (ODP‐TH). This protein hybrid framework encapsulates the photosensitizer protoporphyrin IX (PpIX) and chemotherapeutic agent doxorubicin (Dox), which are attached by a glutathione‐responsive disulfide bond. Mechanistically, ODP‐TH crosses the blood–brain barrier (BBB) and specifically aggregated in hypoxic tumors via protein homology recognition. Oxygen and encapsulated drugs ultimately promote a therapeutic effect by down‐regulating the abundance of multidrug resistance gene 1 (MDR1) and hypoxia‐inducible factor‐1‐*α* (HIF‐1*α*). The results reveal that ODP‐TH achieves oxygen transport and protein homology recognition in the hypoxic tumor occupation. Indeed, compared with traditional photodynamic chemotherapy, ODP‐TH achieves a more efficient tumor‐inhibiting effect. This study not only overcomes the hypoxia‐related inhibition in combination therapy by targeted oxygen transport but also achieves an effective treatment of multiple tumors, such as breast cancer and glioma, providing a new concept for the construction of a promising multi‐functional targeted and intensive anti‐tumor nanoplatform.

## Graphical abstract

A new multi‐functional protein hybrid nanoplatform is designed, consisting of transferrin as the multicategory solid tumor recognizer and hemoglobin for oxygen supply. This study not only overcomes the hypoxia‐related inhibition in combination therapy by targeted oxygen transport but also achieves effective treatment of multiple tumors, providing a new concept for the construction of a promising multi‐functional targeted anti‐tumor nanoplatform.

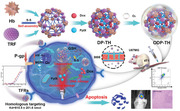

## 1 Introduction

The tumor microenvironment (TME) has many physical and chemical characteristics, such as low oxygen, low pH, high interstitial fluid and high blood vessel permeability, that differ from normal physiological conditions.^[^ ^1^ ^]^ Among these characteristics, a hypoxic environment stands out as being closely related to tumor growth, metastasis, and poor prognosis. Uncontrolled proliferation and abnormal development of internal blood vessels determine the characteristics of tumor hypermetabolism. The high metabolism of tumor cells depletes the internal oxygen, which in turn enables these cells to have a screening effect and further enhances the degree of malignancy of the tumor.^[^ ^2^ ^]^ In recent years, PDT, which kills tumor cells by producing cytotoxic singlet oxygen, combined with laser irradiation of a photosensitizer inside the lesion, has been widely studied for its low toxicity, non‐invasiveness and immune‐activating advantages.^[^ ^3^ ^]^ However, the hypoxic TME limits the production of cytotoxic singlet oxygen during PDT. Moreover, PDT further exhausts the small amount of oxygen present in the environment, thereby causing the treatment to enter into a vicious cycle.^[^ ^4^ ^]^ Traditional chemotherapy drugs, such as Dox, are often criticized for their low targeting and high toxicity, and large doses of these drugs cause irreversible damage.^[^ ^5^ ^]^ It has also been reported that multidrug resistance gene 1 (MDR1) in the hypoxic TME induces the expression of a P‐glucose protein, leading to insensitivity of tumor cells to chemotherapeutic agents.^[^ ^6^ ^]^

Given that a hypoxic environment hinders the effectiveness of traditional therapy, increasing attention is being paid to employ methods to improve the tumor hypoxia microenvironment, such as hydrogen peroxide nanocarriers, Fenton‐like reactions in tumors, manganese dioxide nanosystems and hyperbaric oxygen therapy. These methods solve the problem of hypoxia by carrying oxygen externally or internally.^[^ ^7^ ^]^ Hemoglobin (Hb) is a protein encapsulated in red blood cells that is widely found in vertebrates. One Hb molecule can carry up to four oxygen molecules and release them in hypoxic regions, which can be employed to solve the problem of tissue oxygen supply.^[^ ^8^ ^]^ Based on its oxygen transport capacity and biosafety, the application of Hb in the development of new biomedical nanoplatforms is promising.^[^ ^9^ ^]^ Although Hb has good oxygen transport capacity, there are many hypoxic tissues in the body. The precise transport of nanoparticles into the hypoxic TME is an emerging topic.^[^ ^10^ ^]^

BBB is a physiological barrier, mainly composed of glial cells and capillaries, that separates brain tissue from other tissues. Few liposoluble drugs can cross the BBB through free diffusion,^[^ ^11^ ^]^ and thus, at present, surgery is the main treatment for intracranial tumors such as gliomas. However, most surgical treatments are based on the principle of functional protection and cannot achieve total tumor resection. Therefore, postoperative adjuvant brain administration is also crucial for tumor treatment. Currently, an increasing number of nanoplatforms are being aimed at brain‐targeted drug delivery, in which the nanoplatform with receptor‐mediated transport (RMT) as the cross‐BBB mechanism has better application prospects.^[^ ^12^ ^]^ The application of RMT in the development of drug delivery nanoplatforms is a research hotspot. TRF, an iron‐carrier protein, is widely present in human blood. TRF can recognize and bind to transferrin receptors (TFRs) on the cell membrane surface to complete iron transport in cells through the blood circulation.^[^ ^13^ ^]^ Compared with normal cell membranes, tumor and brain capillary endothelial cell membranes contain significantly higher amounts of TFRs, which provide a greater possibility for recognition and binding with TRF.^[^ ^14^ ^]^ Taking advantage of this feature, a drug delivery nanoplatform targeting tumors across the BBB based on the homologous recognition of TRF and TFRs is highly promising.

**Scheme 1:**
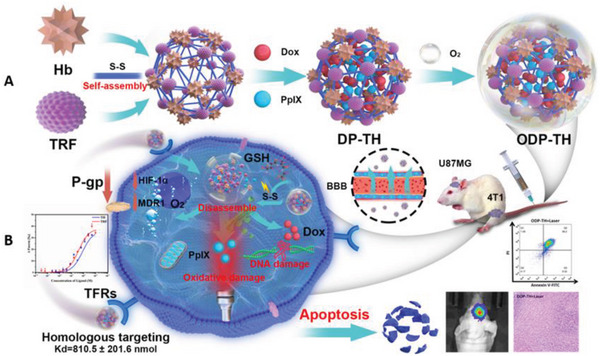
*^a^* The synthesis and intracellular mechanisms of ODP‐TH: A) synthesis of ODP‐TH. DP‐TH was fabricated with internal Dox & PpIX and external TRF & Hb, and then was equipped with oxygen to constructed ODP‐TH. B) The function of ODP‐TH intracellular, including disassemblage of ODP‐TH, synergetic photodynamic‐chemo therapy effectiveness and the inhibition of oxygen‐related markers.

Inspired by this research foundation and considering the necessity of combination therapy,^[^ ^15^ ^]^ in this study, an integrated diagnosis and treatment nanoplatform of PDT combined with chemotherapy coated with a protein hybrid shell was innovatively developed. TRF and Hb were designed for specific hypoxia‐targeting and O_2_ release, respectively, and together formed a protein hybrid via disulfide bonds. Specifically, a multifunctional nanoplatform was formed by encapsulating the photosensitizer PpIX and chemotherapy drug Dox in the hybrid protein shell (ODP‐TH) (**Scheme** **1**). Our results revealed that ODP‐TH successfully crossed the BBB and was absorbed by different types of cancer cells both accurately and rapidly, owing to its excellent homologous protein recognition capacity. Thereafter, the HbO_2_ present in the nanoshell spontaneously and continuously dissociated oxygen in the hypoxic TME, alleviating the intractable hypoxia in the tumor. Our designed strategy not only guarantees the supply of oxygen for PDT but also inhibits MDR1 and HIF‐1‐*α* expression. Overexpression of glutathione (GSH) in the TME breaks the disulfide bond in ODP‐TH, disintegrating the nanoparticle structure and releasing the encapsulated drugs. The ODP‐TH protein hybrid nanoparticle was characterized by morphology, protein composition analysis, and spectroscopy. Strategical evaluations were carried out to confirm the anti‐tumor effects of ODP‐TH both in vivo and in vitro. Further analysis indicated that the hypoxia phenomenon in the TME was successfully overcome by in vitro loading oxygen, providing support for PDT and chemotherapy. In addition, homologous targeting of the TRF markers boosted the effectiveness of both PDT and chemotherapy. Notably, the long‐term retention effect of drugs based on ODP‐TH in tumors provides a feasible way for low‐dose and high‐efficiency treatment. Collectively, this study developed a new multifunctional synergistic anticancer therapy that specifically targets against hypoxic TME.

## 2 Results and Discussion

### 2.1 Synthesis and Characterization of the ODP‐TH Nanoplatform

**Figure 1:**
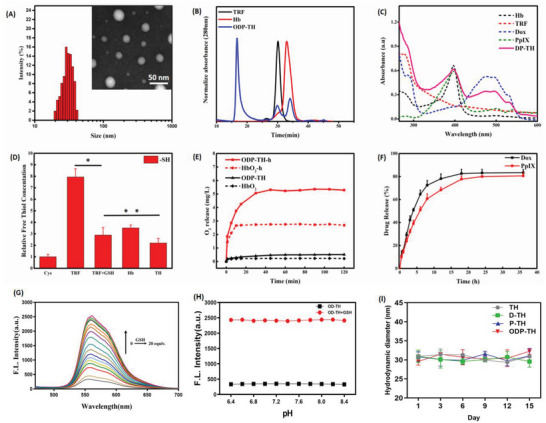
A) Size distribution and TEM of ODP‐TH; B) SEC of TRF, Hb, and TH nano shell. C) UV–vis absorption spectroscopy of Hb, TRF, Dox, PpIX, and DP‐TH. D) Ratio of free sulfhydryl concentration in different groups. E) Oxygen release curves of ODP‐TH and HbO_2_ under normoxia and hypoxia conditions. F) Drug release curves of ODP‐TH incubated with GSH (5 mm) in different times. Fluorescence intensity curve of Dox encapsulated in D‐TH treated with different concentrations of GSH (G) and same concentration of GSH (5 mm) at the pH values from 6.6 to 8.4 at 25 °C (H). I) The hydrodynamic diameter curves of TH, D‐TH, P‐TH and ODP‐TH dispersed in PBS on D1, D3, D6, D9, D12 and D15 at 25 °C. Mean ± SD, *n* = 3. *p*‐value were indicated by * (*p* < 0.05) and ** (*p* < 0.01)

The detailed synthetic process for ODP‐TH is illustrated in Scheme 1A. Transmission electron microscopy and dynamic light scattering (DLS) techniques revealed that ODP‐TH comprises a regular smooth spherical shape with a diameter of 30.2 ± 1.2 nm. To investigate whether ODP‐TH was successfully synthesized in the process indicated in Scheme 1A, we employed size exclusion chromatography. The results showed that the mass of ODP‐TH was significantly higher than that of pristine TRF and Hb, indicating a new substance comprising both TRF and Hb was formed. Small amounts of pristine TRF and Hb were also detected in the ODP‐TH solution (**Figure** **1**B). In addition, the UV–vis absorption spectrum of ODP‐TH presented characteristic absorption peaks of TRF and Hb together with distinct peaks of Dox and PpIX (Figure 1C). Next, we speculated that treatment with excessive GSH might crosslink TRF and Hb via disulfide bonds. To test this possibility, we used a previously reported method by measuring the concentration of free sulfhydryl to determine whether disulfide bonds could be formed.^[^ ^16^ ^]^ The concentration of the free sulfhydryl groups in the cysteine solution was used as a benchmark. We found that the concentration of free sulfhydryl groups in the TH nano‐shell solution was significantly lower than that of either the Hb or TRF groups (Figure 1D), suggesting that the two groups were crosslinked by disulfide bonds. Therefore, we concluded that ODP‐TH was successfully synthesized.

### 2.2 Physical and Chemical Properties of the ODP‐TH Nanoplatform

The main focus of this study was the oxygen transport capability of ODP‐TH. As shown in Figure 1E, in the hypoxic PBS solution containing the same concentration of Hb, the oxygen release of ODP‐TH was almost twice that of HbO_2_. This high release capability can be attributed to the stable cross‐linking of TRF and Hb through disulfide bonding, which improves the stability of the oxygen molecules. Conversely, this phenomenon was not observed in the PBS solution with a normal oxygen content under the same conditions (Figure 1E). Based on these observations, we concluded that ODP‐TH would have a better oxygen‐releasing capability and could solve the problem of hypoxia in the TME. Furthermore, we studied the drug release pattern from ODP‐TH. As shown in Figure 1F, ODP‐TH released Dox and PpIX when treated with excess GSH over time. More specifically, 78.1% Dox and 60.6% PpIX were released in the first 12 h of testing. We observed similar kinetics using TEM, wherein the edge of ODP‐TH became blurred, and the shape changed irregularly when incubated with the same GSH concentration in the TME. Finally, the overall morphology of ODP‐TH was significantly disintegrated at the 24 h timepoint (Figure S1, Supporting Information). Meanwhile, the effect of increasing equivalent GSH on drug release of single loaded D‐TH was detected (Figure 1G), the fluorescence intensity positively related to the amount of Dox release increased rapidly, and the drug release rate tended to slow down to a plateau when GSH concentration was elevated to 14 equivalents. There is also no significant drug release detected in the near physiological environment range of pH 6.4 to 8.4 with GSH absence (Figure 1H), which indicated GSH is the trigger of our nanoplatform disassembling. The stability of our nanoplatform was also evaluated. Nanoparticles were stored in PBS at 25 °C for 15 days, and morphological records were made by transmission electron microscopy at D1, D3, D6, D9, D12 and D15. As shown in the Figure S2, Supporting Information, the morphology of nanoparticles remains smooth and spherical over time which indicated high stability. Additionally, the hydrodynamic diameter of nanoplatform at different timepoints stored in PBS was also recorded. No matter that the nanoparticles are unloaded, single‐loaded or dual‐loaded, the hydrodynamic diameter did not change significantly within 1 to 15 days, which indicated the satisfying stability of this nanoplatform (Figure 1I). Similarly, the stable Zeta potential also supported the above view (Figure S3, Supporting Information).

### 2.3 Recognition Capacity of ODP‐TH for TFRs

**Figure 2:**
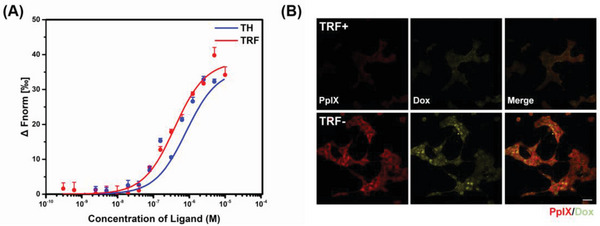
A) MST assay of binding affinity between TFRs and TH nano shell, TRF. B) CLSM images of ODP‐TH uptake in 4T1 cells treated with TRF (500 µm) for 4 h and unpretreated 4T1 cells. Mean ± SD, *n* = 3. Scale bar = 100 µm.

Given that TRF is a ligand protein that can be specifically recognized by TFR, we next proceeded to verify whether the TFRs are a direct target for TH nanoshells. By performing microscale thermophoresis assay, we observed that TFRs bind the TH nano shells and TRF with analogous dissociation constants of 810.5 ± 201.6 and 385.3 ± 66.0 nmol, respectively (**Figure** **2**A). These results suggested that the nanoplatforms possess excellent protein homologous targeting ability, which provides mechanistic support for the subsequent anti‐tumor test. In addition, in 4T1 cells pre‐incubated with excess TRF, the uptake of ODP‐TH was markedly reduced. As shown in the CLSM images in Figure 2B, the fluorescence intensities of PpIX and Dox in the TRF+ group are significantly weaker than that in the TRF‐group. This phenomenon is most likely due to the large number of TFRs pre‐occupied by TRF, resulting in the inability of ODP‐TH to homophilically recognize the TFRs on the 4T1 cells, such that a large amount of free ODP‐TH outside the cell cannot enter the cell. In contrast, the untreated 4T1 cells retained an excellent uptake capacity for ODP‐TH, the similar phenomena also occurred in U87MG cells (Figure S4, Supporting Information).

### 2.4 Cytotoxicity and Oxygen Transport Capacity of the ODP‐TH In Vitro

**Figure 3:**
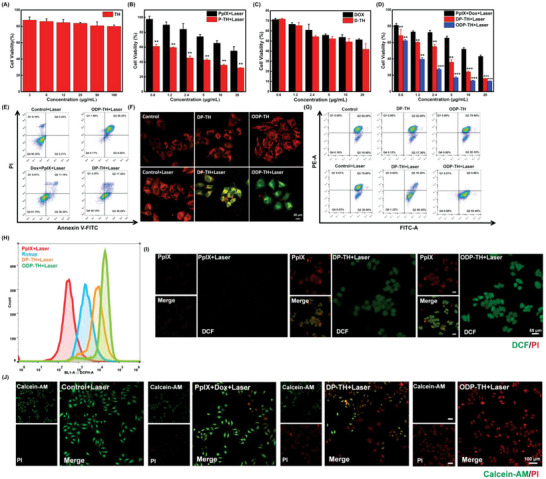
The cell viability of 4T1 cells treated with different concentrations of A) TH nano shell for 24 h. B) free PpIX and P‐TH for 4 h and then lasered for 5 min. C) Free Dox and D‐TH for 24 h. D) Treated with different concentrations of PpIX + Dox, DP‐TH, and ODP‐TH for 12 h and following with 5 min irradiation. E) Flow cytometry analysis of 4T1 cells apoptosis incubated with DP‐TH and ODP‐TH for 12 h following with 5 min irradiation. CLSM images (F) and flow cytometry analysis (G) of JC‐1 for 4T1 cells incubated with DP‐TH and ODP‐TH for 4 h and then lasered for 10 min or not. Red is polymeric JC‐1, green is monomeric JC‐1. Flow cytometry analysis (H) and CLSM images of DCF fluorescence (I) for 4T1 cells incubated with PpIX, P‐TH and OP‐TH for 4 h, then lasered for 5 min or not. J) Fluorescence images of 4T1 cells incubated with PpIX + Dox, DP‐TH, and ODP‐TH for 4 h and then lasered for 5 min or not. Green is Calcein‐AM, red is propidium iodide (PI). Mean ± SD, *n* = 3, *p*‐value were indicated by ** (*p* < 0.01) and *** (*p* < 0.001).

The low toxicity of nanomaterials is the premise for their safety both in vivo and in vitro. First, we co‐incubated mouse breast cancer 4T1 cells with different TH nanoshell concentrations without drug encapsulation (**Figure** **3**A). As expected, the TH nanoshell displayed a satisfactory biological low‐toxicity performance. After treatment with different concentrations of TH nanoshells for 24 h, ≈80% of the treated 4T1 cells was still alive which provided a safety guarantee for our next series of in vitro effect studies of this nanoplatform. Second, we explored the performance of single encapsulated nanoparticles, P‐TH and D‐TH, for in vitro anti‐tumor therapy (Figure 3B,C). Unexpectedly, the P‐TH + Laser group demonstrated better anti‐tumor activity than the PpIX group, and similar results were observed in the D‐TH and Dox groups. This suggests that the anti‐tumor activity of the drug is improved after encapsulation by nanomaterials, which is probably due to the targeting effect of TRF that promotes the drug concentration in 4T1 cells and stimulates the effect of PDT and chemotherapy. We focused on whether oxygen carried by ODP‐TH could improve its curative effect. Figure 3D illustrates that the killing effect of the ODP‐TH + Laser group on 4T1 cells was significantly better than that of the DP‐TH and PpIX + Dox + Laser groups. This suggested that ODP‐TH alleviated the hypoxic state in the TME and enhanced the effects of the drugs. Similar results were observed in the apoptosis experiments (Figure 3E). Under the premise of the same dosage, the proportion of apoptotic cells in the ODP‐TH + Laser group was fivefold greater than that in the DP‐TH + Laser group and eightfold greater than that in the PpIX + Dox + laser group, which proved that ODP‐TH possesses anti‐tumor activity. The mitochondrial membrane potential changes irreversibly in the early stage of apoptosis, and the JC‐1 polymers and monomers can be detected by lasers with different wavelengths. Based on this feature, we used the mitochondrial fluorescence probe JC‐1 to determine whether ODP‐TH is more toxic to 4T1 cells than to the other groups. As shown in the merged CLSM images in Figure 3F, the JC‐1 monomer occupies a more dominant position in the ODP + Laser group than in the other laser groups; meanwhile, this trend is not observed in the groups without laser. This indicates that the PDT in the ODP‐TH + Laser group produces more cytotoxic singlet oxygen with oxygen introduction, and the enhanced targeting causes more Dox to accumulate in the cancer cells. This, in turn, causes depolarization of the mitochondrial membrane potential. The same conclusion was obtained using flow cytometry (Figure 3G). Cytotoxic singlet oxygen production is critical for PDT, however, the hypoxic environment in tumors limits the PDT efficacy. To explore whether ODP‐TH could successfully supply enough oxygen to cancer cells, we selected flow cytometry and CLSM to detect the influence of ODP‐TH on reactive oxygen species (ROS) production. As shown in Figure 3H, the fluorescence of DCF in the ODP‐TH group was the strongest, exceeding that of the positive control group (Rosup). From the CLSM images (Figure 3I), the green DCF fluorescence was also the brightest in the ODP‐TH group, owing to the unloading of the DP‐TH oxygen. These results indicated that ODP successfully released oxygen and produced a large amount of ROS in the cancer cells, which is a prerequisite for PDT. Simultaneously, the Calcein‐AM/PI Double Stain Kit was used to support this view. As expected, the 4T1 cells, which were stained red by PI, were more prominent in the ODP‐TH + Laser group than in the other irradiated groups (Figure 3J).

### 2.5 Cellular Uptake and Inhibition of Hypoxia‐Related Gene Expression of the ODP‐TH Nanoplatform

**Figure 4:**
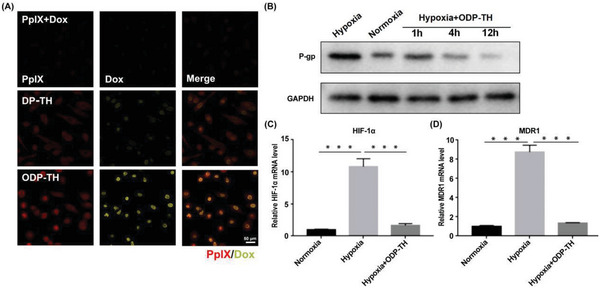
A) CLSM images of uptake for hypoxia 4T1 cells after incubation with PpIX + Dox, DP‐TH and ODP‐TH for 12 h. B–D) WB and qRT‐PCR analysis of P‐gp (B), HIF‐1*α* (C), and MDR1 (D) expression of 4T1 cell in different states treated with ODP‐TH for different time. Mean ± SD, *n* = 3. *p*‐values were indicated by *** (*p* < 0.001).

According to the results of the in vitro anti‐tumor effect, PpIX and Dox were precisely transported to the 4T1 cells due to targeting of the TRF and EPR effects of the nanoparticles. However, as shown in **Figure** **4**A, Dox and PpIX uptake in the ODP‐TH group containing the same amount of drug was significantly higher than that in the DP‐TH group. This phenomenon is probably caused by P‐gp, which is encoded by the multidrug resistance 1 gene could pump various chemo drugs out of tumor cells. The hypoxia of the TMR leads to P‐gp overexpression, which excludes the Dox transported by DP‐TH from the cancer cells and thus affects the performance of chemotherapy drugs. To verify this inference, we studied the P‐gp expression in 4T1 cells treated with ODP‐TH for different durations (1 to 12 h). Western blotting (WB) results showed that P‐gp protein expression in 4T1 cells co‐incubated with ODP‐TH was lower than that in 4T1 cells under hypoxic conditions. Moreover, P‐gp expression in the 4T1 cells was significantly downregulated with the extension of incubation time and became almost the same as that of normoxic cells within ≈4 h (Figure 4B and Figure S5, Supporting Information). Meanwhile, MDR1 and hypoxia‐inducible factor‐1‐*α* (HIF‐1*α*) expression in the 4T1 cells treated with ODP‐TH was also detected by real‐time quantitative polymerase chain reaction (RT‐qPCR). Following the above results, the mRNA expression levels of both decreased by more than 80% compared with that of the hypoxia group and were very close to the normoxia group after 2 h of incubation with ODP‐TH (Figure 4C). These results confirmed that ODP‐TH significantly alleviated the hypoxic environment in tumors and mitigated drug resistance in hypoxic cancer cells by downregulating P‐gp. Based on the above findings, we expect ODP‐TH to work well in in vivo studies.

### 2.6 In Vivo Biosafety and Distribution of the ODP‐TH Nanoplatform

**Figure 5:**
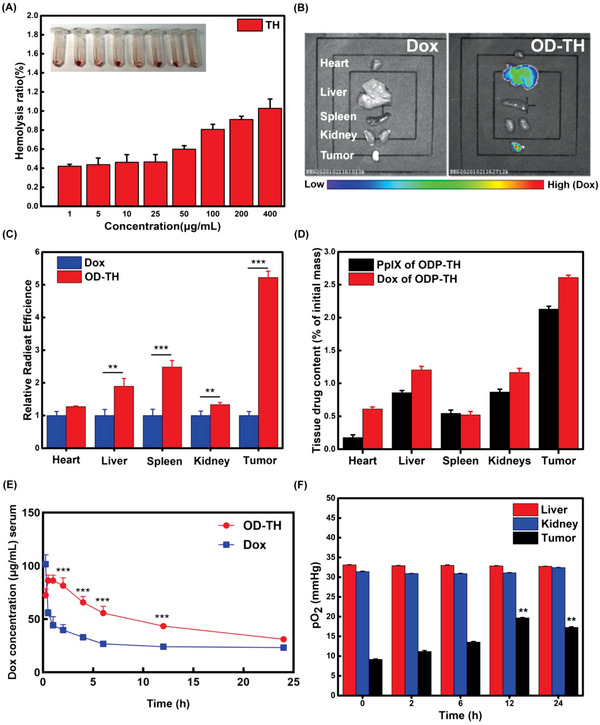
A) Hemolysis rate of TH nano shell at different concentrations. B) In vivo fluorescence image of major organs and solid tumor 48 h after injection of OD‐TH. C) Relative fluorescence histogram of major organs and tumor 48 h after injection of Dox and OD‐TH. D) Distribution of PpIX and Dox in major organs (heart, liver, kidney, spleen) and solid tumor 48 h after injection of ODP‐TH. E) Pharmacokinetics analysis of single loaded OD‐TH and free Dox in serum of 4T1 tumor bearing mice at 0.25, 0.5, 1, 2, 4, 6, 12, and 24 h after injection, i.v., with the dosage of 10 mg kg^−1^. F) Changes of pO_2_ in liver, kidney, and tumor of ODP‐TH treated mice at different time. Mean ± SD, *n* = 3. *p*‐values were indicated by ** (*p* < 0.01) and *** (*p* < 0.001).

Encouraged by the results of the previous in vitro research, we proceeded to explore the role of this nanoplatform in vivo. 4T1 tumor‐bearing BALB/c nu mice were used as the experimental subjects. Initially, the hemolysis rate test was used to evaluate the biological safety of the TH nanoshells in vivo. As shown in **Figure** **5**A, the TH nanoshells exhibited a negligible hemolysis rate at a concentration of 400 µg mL^−1^, indicating satisfactory biocompatibility. Subsequently, small‐animal imaging technology was used to observe the function of the nanoplatforms in vivo. To avoid fluorescence interference by PpIX, the mice were intravenously injected with OD‐TH. With time, there was a rapid accumulation of fluorescence at the tumor site, which could be clearly distinguished from that of normal tissues. Notably, the fluorescence intensity reached a maximum at 12 h, highlighting the advantages of the prominent rapid aggregation of ODP‐TH (Figures S6 and S7, Supporting Information). In addition, there was prominent fluorescence in the tumor site at the fourth hour, indicating that a large amount of Dox was released rapidly from the nanoshell and remained at a certain concentration for up to 48 h. Such a long‐term retention effect would greatly improve the therapeutic effect. Meanwhile, we collected fluorescence images of the metabolic organs and solid tumors from the mice subjects 48 h after injection. Consequently, tumor tissue fluorescence was still significantly more substantial than that in other organs (Figure 5B). Similar results are shown in Figure S8, Supporting Information. The fluorescence intensity of the tumor and major organs in the OD‐TH group was stronger than that in the Dox group 24 h after intravenous injection of the same amount of Dox (Figure 5C). Moreover, the fluorescence intensity of the tumors in the OD‐TH group was more than fivefold greater than that in the Dox group. Finally, the quantitative fluorescence method was used to determine the PpIX and Dox distributions in the major organs and tumors of mice. As expected, the PpIX and Dox distributions in the tumors were more than double those in the metabolic organs (Figure 5D). Notably, the cardiotoxicity of Dox may lead to more accumulation in the heart.^[^ ^17^ ^]^ Pharmacokinetic experiments were performed to explore the metabolism of ODP‐TH in the blood circulation of mice. As exhibited in pharmacokinetic curves (Figure 5E), it was observed that free Dox rapidly accumulates in organs especially the heart for its non‐targeting resulting in a rapid drop of serum drug concentration in the first one hour after injection by more than 50%. In contrast, the concentration of Dox in OD‐TH group increased slightly in the first hour after injection, which is probably attributable to a small amount of Dox released in the tumor entering into the peripheral blood, and the release speed being faster than elimination. As time increased, more and more nanoparticles were eliminated, and the concentration of serum Dox in the OD‐TH group gradually decreased and was basically the same as that in the free Dox group at 24th hour after injection. Our nanoplatform exhibited sustained release performance, extending the effect time of drugs and providing practical support for improving therapeutic efficiency. Moreover, all the results of our designed nanoplatform displayed the excellent function of targeting solid tumors and provide a valuable reference for the in vitro diagnosis of solid tumors.

### 2.7 In Vivo Oxygen Transport of ODP‐TH

We supposed that the study of the changes in the oxygen distribution in mice injected with ODP‐TH would reveal the targeting oxygen transport performance of the ODP‐TH nanoplatform more directly. Therefore, the pO_2_ levels in the liver, kidney, and tumors of the post‐injection mice were measured to evaluate the oxygen transport capacity of the nanoplatform. As expected, the results showed that there was no significant change in the pO_2_ levels of the liver and kidney in the mice before and after injection. In contrast, the pO_2_ level of the tumor reached the highest value of 19.7 mmHg at 12 h, which is 10.5 mmHg higher than that before injection. Moreover, after 12 h, the tumor pO_2_ level began to decline with the increase in time, confirming the observations made from the in vivo fluorescence imaging (Figure 5F). Consequently, ODP‐TH transported and released oxygen into the solid tumor and maintained a high oxygen concentration within 24 h. In comparison, ODP‐TH always maintains a firm structure and rarely dissociates oxygen in other metabolic organs with normal pO_2_ levels.

### 2.8 Production of Singlet Oxygen and Hypoxia‐Related Immunofluorescence Imaging of the ODP‐TH

**Figure 6:**
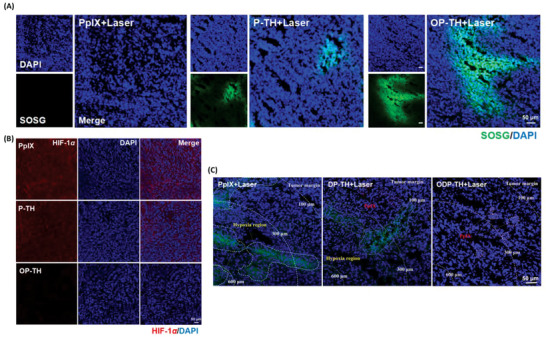
A) CLSM images of SOSG (green) probe evaluating the formation of singlet oxygen after treated with PpIX, P‐TH and OP‐TH followed by 650 nm laser for 10 min (100 mW cm^−2^). B) Ex vivo immunofluorescence images of HIF‐1*α* (red) in tumor of different groups. C) Ex vivo immunofluorescence images of hypoxia area (green) in tumor stained by Hypoxyprobe.

A large amount of ROS can be produced during PDT, and singlet oxygen is considered to play a crucial role in the photodynamic effect. To evaluate the PDT effect of the nanoplatforms, singlet oxygen generation was tested in vivo using singlet oxygen sensor green (SOSG) 4 h post‐injection. As illustrated in **Figure** **6**A, there was no distinct singlet oxygen generation in the PpIX group, owing to insufficient oxygen supply and negligible photosensitizer in the tumor tissue. Notably, the OP‐TH group provided an interesting contrast to the P‐TH group, with larger green areas indicating more singlet oxygen generation, and thus, a greater oxygen supply. The accumulation of PpIX and sufficient oxygen supply in the tumor can be attributed to the excellent drug target delivery and sustainable oxygen release in the hypoxic TME achieved by the nanoplatforms. The immunofluorescence results also support the above viewpoint. The bright red region in the PpIX and P‐TH groups indicated that HIF‐1‐*α* was still widely expressed in the tumor tissue compared to that in the ODP‐TH group. The darkness suggested down‐regulation of HIF‐1‐*α* expression (Figure 6B). The oxygen production efficiency of OP‐TH was further detected by a Hypoxyprobe that could locate the hypoxic area in the tumor tissue. The level of hypoxia is far greater in the tumor tissue than on the tumor surface, and our focus was to determine whether the ODP‐TH nanoplatform can help resolve the hypoxia that is present in the deep tumor tissue. As shown in Figure 6C, the green area, which indicates extremely deoxygenated tissue in the P‐TH group, was sparser than that in the PpIX group but disappeared in the OP‐TH group. These results more intuitively showed that OP‐TH provided sufficient oxygen supply in the hypoxic tumor tissue and that the hypoxic state was greatly alleviated. Notably, we observed abundant red signals in the P‐TH and OP‐TH groups, revealing the presence of large quantities of PpIX and thus confirming the accurate drug delivery performance of the nanoplatforms.

### 2.9 Evaluation of the Synergistic Therapeutic Effect of the ODP‐TH Nanoplatform In Vivo

**Figure 7:**
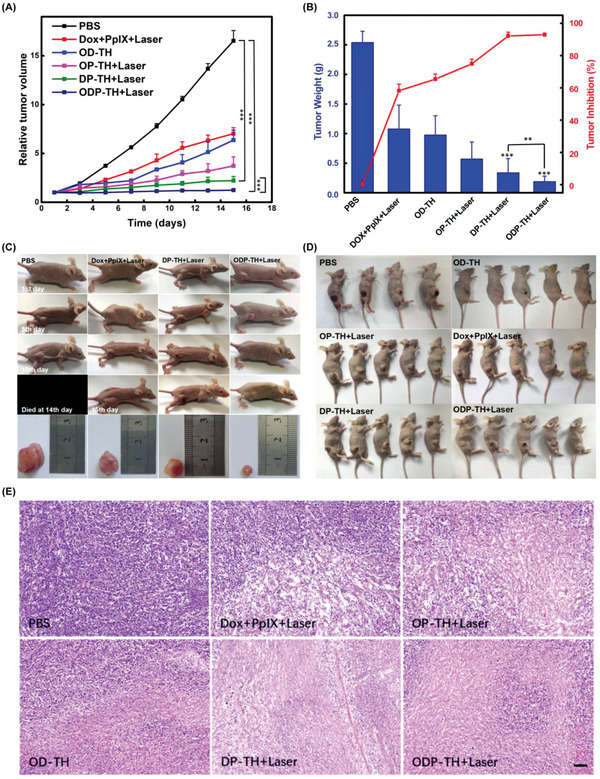
Therapeutic effect of ODP‐TH combined with PDT and chemotherapy on mice. A) 4T1 tumor volume changes curves of different groups within 15 days after administration. B) Tumor weight and inhibition rate in mice after 15 days after treatment. C) Photographs of four representative groups of mice and corresponding dissected tumor 15 days after treatment. D) Photographs of six representative groups of mice at the termination of administration. E) Six representative HE staining images of dissected tumor (100×). Mean ± SD, *n* = 5. *p*‐values were indicated by *** (*p* < 0.001), scale bar = 100 µm.

In the subsequent evaluation of the ODP‐TH efficacy, eight‐week‐old female BALB/c nu mice bearing 4T1 tumors with uniform body weight were used as in vivo anti‐tumor experimental animals. The mice were randomly divided into 13 groups: 1) PBS; 2) PpIX + Laser; 3) Dox; 4) PpIX + Dox + Laser; 5) OP‐TH; 6) OP‐TH + Laser; 7) P‐TH + Laser; 8) D‐TH; 9) OD‐TH; 10) DP‐TH; 11) DP‐TH + Laser; 12) ODP‐TH; and 13) ODP‐TH + Laser. When the tumor volume reached about 100 mm^3^ and mice were treated every 3 days. As shown in **Figure** **7**A, there was no inhibitory effect on rapid tumor growth in the PBS group during treatment. Similarly, the tumor growth inhibition effect was unsatisfactory in the PpIX + Dox + Laser group, owing to the rapid clearance of low‐targeted chemotherapeutic drugs and inefficient PDT in the hypoxic regions. In contrast, the tumor in the OD‐TH group was significantly suppressed compared to that in the Dox and D‐TH groups because of the higher doses of chemotherapy drugs in the tumor tissue and homologous targeting of TRF. Similarly, tumors in the OP‐TH + Laser group grew at a much slower rate than those in the PpIX + Laser and P‐TH + Laser groups, indicating a significantly improved PDT effect for the relief of hypoxia (Figure S9, Supporting Information). Notably, the ODP‐TH + Laser group, which benefited from the synergetic enhancement of both chemotherapy and PDT, exhibited the best anti‐tumor results among all the studied groups (Figure 7A and Figure S10, Supporting Information). The results shown in Figure 7B and Figure S11, Supporting Information, also affirmed the robust enhancement of the therapeutic effect with the administration of multifunctional ODP‐TH. Additionally, four representative groups were selected to record the tumor morphological changes before and after administration to verify the excellent anti‐tumor effectiveness of ODP‐TH (Figure 7C). All groups of mice and tumor dissections were recorded on the 15th day post injection (Figure 7D and Figure S12, Supporting Information). Tumor growth of mice in the ODP‐TH + Laser group was significantly inhibited under the same initial conditions. As shown in Figure S13, Supporting Information, the weight of all the studied mice changed negligibly during the experiment, which suggests mild experimental conditions and a low general toxicity of our experimental samples. Furthermore, the remarkable apoptosis of the 4T1 tumors treated with ODP‐TH + Laser is clearly shown in the hematoxylin and eosin (HE) images (Figure 7E and Figure S14, Supporting Information). WB and RT‐qPCR were also performed to evaluate the expression of oxygen related proteins in 4T1 tumor, the results were consistent with that in 4T1 cells tests (Figure S15, Supporting Information). To detect whether the nanoplatform caused damage to the major organs of the mice, HE slices of major organs and ALT/AST/CRE/BUN indices in the mice were evaluated after treatment (Figures S16 and S17, Supporting Information). The results of the ODP‐TH + Laser group showed that these indicators were all within the normal physiological range, which again verified the good biosafety of the nanoplatform.

### 2.10 Evaluation of the ODP‐TH Nanoplatform on Crossing the BBB both In Vivo and In Vitro

**Figure 8:**
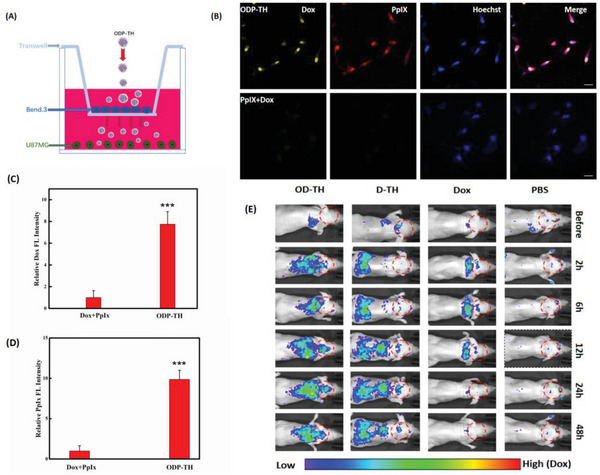
Evaluation of the ability of nanometers to cross the BBB. A) Simulation of the BBB model in vitro. B) The CLSM images of U87MG cells incubated with ODP‐TH, PpIX, and Dox for 4 h in transwell and fluorescence semi‐quantitative statistics (C,D). Fluorescence of Dox in orthotopic glioma model at different time (E). Mean ± SD, *n* = 3. *p*‐values were indicated by *** (*p* < 0.001). Scale bar = 20 µm.

To explore the BBB‐crossing capacity of the as‐produced ODP‐TH, we established a BBB model using a Transwell chamber in vitro and adopted a real‐time imaging method to detect drug delivery (**Figure** **8**A). The experimental group was administered ODP‐TH, while the control group was administered 5 µg mL^−1^ concentrations of free Dox and PpIX. As shown in Figure 8B, after 4 h of administration, we observed marked Dox and PpIX fluorescence in the ODP‐TH‐administered group. In contrast, Dox and PpIX fluorescence was obscured in the control group, except for the Hoechst fluorescence‐stained living cells. After semi‐quantitative analysis, we calculated the average fluorescence quantities of Dox and PpIX in the U87MG cells to represent the amount of drugs transported across the BBB to the CNS. The results showed that in the ODP‐TH group, the U87MG cells rapidly ingested the nanoparticles and released large amounts of Dox and PpIX in the cells. The average Dox and PpIX fluorescence intensities of the experimental group were respectively 7.9 and 9.9 times greater than those of the control group (Figure 8C,D). The reason for this significant uptake difference is that the TRF on the shell of the ODP‐TH nanoplatform can interact with the overexpressed TFRs in the U87MG cells and mediate transmembrane transport. Numerous nanoparticles successfully crossed the BBB and released Dox mediated by GSH in the U87MG cells. In comparison, these drug molecules failed to cross the BBB to be absorbed by the tumor cells through simple diffusion. We selected an in vivo imaging system to evaluate the performance of the nanoplatform across the BBB in animal models. As shown in Figure 8E, the Dox fluorescence signal of the OD‐TH group was stronger than that of the free Dox group 2 h after administration, which was 4.21 times that of its average fluorescence intensity after statistical analysis. With the passage of time, the intensity of the fluorescence signal in the brain region of the OD‐TH group was relatively high, and its fluorescence intensity was 9.54 times that of the Dox group after 48 h. There was no significant drug accumulation in the brains of the Dox group, indicating that the single free Dox molecules were blocked by the BBB. A small amount of Dox accumulated in D‐TH within 48 h. The BLI and FLI colocalization image of the dissected brain after 48 h of treatment also supported the living imaging results (Figure S18, Supporting Information). In summary, we demonstrated that ODP‐TH can successfully penetrate the BBB and aggregate in the intracranial tumor area. From these results, we can conclude that the ODP‐TH nanoplatform has an excellent ability to penetrate the BBB, providing in vitro experimental support for its application prospect in the treatment of CNS diseases.

**Figure 9:**
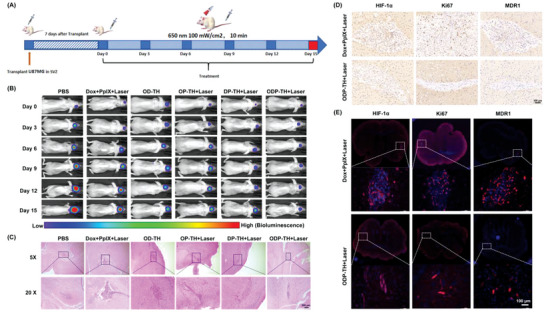
Administration process of in orthotopic glioma mice (A). Bioluminescence imaging of mice heads in different treatment groups (B). Enhanced effectiveness evaluation of ODP‐TH on the orthotopic glioma model. C) HE stained brain slices, glioma tissues were circled by dotted line(10×). D) Immunohistochemistry images of glioma tissues. (30×). E) Immunofluorescence images of glioma tissues stained with HIF‐1*α* antibody (red), Ki‐67 antibody (red), and MDR‐1 antibody (red) (20×). Mean ± SD, *n* = 3.

Luciferase‐labelled U87MG (U87MG‐Luc) cells were implanted into the SVZ region of an eight‐week‐old female BALB/c nu mouse brain for further anti‐orthotopic glioma studies. Ten days after implantation, the mice were randomly divided into six groups. The volumes of all the U87MG tumors were recorded every 3 days by bioluminescence imaging (**Figure** **9**A). The bioluminescence results in Figure 9B revealed that the tumor growth rate of the PBS and Dox + PpIX + laser groups were relatively rapid, while that of the OP‐TH + laser, OD‐TH, DP‐TH + laser and ODP‐TH + laser groups slowed down in varying degrees. The tumor volume of the ODP‐TH + laser group did not change significantly compared to that of the other groups. According to the statistical results of the BLI images during the whole treatment process, compared with the PBS group, the tumor inhibition rate of the ODP‐TH + laser group reached 97.2%, which indicated the excellent tumor inhibition ability of the ODP‐TH nanoplatform. Similar results were observed in the OP‐TH + laser, OD‐TH, and DP‐TH + laser groups to varying degrees (Figure S19, Supporting Information). By monitoring the weight of the experimental mice during treatment, except for the weight loss caused by the growth of malignant tumors in the PBS and Dox + PpIX + laser groups, the weight of mice in the nanoplatform‐treated groups remained at a stable level (Figure S20, Supporting Information). These results demonstrated the high biosafety of the ODP‐TH nanoplatform.

We next analyzed brain slices of the mice treated using the different strategies. As exhibited in Figure 9C, the nucleus of the tumor cells in ODP‐TH + Laser group shrunk or became fragmented. As shown in Figure 9D,E, HIF‐1*α*, Ki‐67, and MDR‐1 expression in the ODP‐TH + Laser group was significantly downregulated compared to that in the control group. Moreover, in the HE‐stained major organs slices, we observed that no pathological changes occurred in the major organs of all the experimental groups (Figure S21, Supporting Information).

## 3 Conclusion

In summary, a multifunctional nanoplatform, namely ODP‐TH, that combines the characteristics of tumor‐targeted aggregation and synergistic therapeutic effects, was fabricated. The nanocomposite possesses better oxygen‐carrying capacity than monomer Hb, and could carry oxygen facilely and automatically release oxygen in the deep hypoxic parts of tumors, significantly alleviating hypoxia in the TME. Additionally, the homologous interactions of the TRF and TFRs target ODP‐TH more precisely than those of NPs, which rely only on the EPR effect on the target tumor. Moreover, drug distribution studies showed that PpIX and Dox encapsulated in ODP‐TH were abundant at the tumor site 48 h post‐injection, which is attributed to the smaller diameter and homologous targeting function of ODP‐TH. Notably, with the support of sufficient oxygen from ODP‐TH, our strategy provides the necessary materials for PDT and significantly alleviates the problem of drug resistance in chemotherapy. Anti‐tumor experiments performed both in vivo and in vitro demonstrated that the anti‐tumor effect of ODP‐TH precedes that of PDT combined with chemotherapy. Therefore, this multifunctional nanoplatform provides an intensive approach and has promising application prospects for cancer treatment.

## 4 Experimental Section

### Materials

Bovine hemoglobin and GSH were purchased from Aladdin (Shanghai, China); transferrin and doxorubicin hydrochloride salt were supplied by MACKLIN (Shanghai, China); PpIX was bought from Energy Chemical (Shanghai, China); ROS detection Kit was obtained from Beyotime (Jiangsu, China); TFRs was purchased from Mei Mian Biotechnology Co., Ltd. (Jiangsu, China)**;** Mouse ALT Kit, BUN Kit, CRE Kit, AST Kit were obtained from Nanjing Jiancheng Bioengineering Institute (Jiangsu, China); Hemoglobin was supported by ToYongBio Tech Inc. (Shanghai, China); Monolith RED‐NHS protein labeling kit were purchased from NanoTemper Technologies GmbH; Hypoxyprobe‐1 plus kit was purchased from Hypoxyprobe Inc.; JC‐1 assay kit, apoptosis assays kit, and Calcein/ PI cell viability assay kit were obtained from keygen biotech (Jiangsu, China).

### Instrument

DLS instrument (Brookhaven 90Plus, USA) was used to detect size distribution. Size exclusion chromatography was analyzed by Protein Purification and Analysis System (AKTA pure, GE, USA) with Superdex 200 10/300 GL (GE, USA). Morphological display was performed by transmission electron microscope (Tecnai 12, Philips). The absorption spectra of the ODP‐TH were administrate by UV–vis spectrometry (UV‐2550, Shimadzu), and the fluorescence spectra was detected by fluorescence spectroscopy (Edinburgh Instruments, FS5 Spectrofluorometer). Zeta potential was measured by Zetasizer Nano Z (MAL1016020, Malvern). In vitro cytotoxicity assays and free thiol measured by enzyme‐labeled instrument (INFINITE 200 PRO, Switzerland). Nanotemper Monolith NT.115 pico instrument (NanoTemper Technologies GmbH) was selected to carry out MST. All the fluorescence imaging photos were taken by CLSM (Leica, SP8). The pO_2_ of liver, kidney, and solid tumor of mice were detected by tissue oxygen partial pressure system (Oxford Optronix, OxyLite). Flow cytometry were performed by acoustic focusing flow cytometry (Thermofisher, Attune NxT). Pharmacokinetics analysis was performed by high performance liquid chromatographic instrument (LC‐20AT, Shimadzu) with InertSustain C18 (5 µm, 250 × 4.6 mm, Shimadzu)

### Synthesis and Characterization of ODP‐TH

TRF (5.0 mg) and GSH (3.0 mg) were bathed in 2 mL ultrapure (UP) water for 1 h at 37 °C. Reaction solution was moved to boiled dialysis bag (3 KD) for 12 h. Hb (1.6 mg), Dox (0.8 mg), and 100 µL of PpIX DMSO solution (8 mg mL^−1^) were added to the mixture and then adjust PH to 8‐8.5 by saturated NaHCO_3_ solution. Next, stirring for 3 h after mixing with absolute ethyl alcohol (3 mL). Thereafter, mixture was moved to a boiled dialysis bag (100 KD) resting for 24 h in UP water. The mixture was centrifuged at 10 000 rounds per minute for 20 min and then the supernatant was removed. The collected precipitate was dispersed with UP water and filtered by a liposome extruder with a 100 nm filter membrane. Finally, the mixture was moved to a lyophilizer for 24 h. The dry powder was DP‐TH, stored at 4 °C. A certain concentration of DP‐TH solution was placed in a three‐way pipe, and then nitrogen and oxygen were introduced respectively for three times at least to synthesize oxygen free DP‐TH and oxygen carrying ODP‐TH.

Size distribution was detected by DLS instrument (Brookhaven 90Plus, USA) at 25 °C. Size exclusion chromatography was analyzed by Protein Purification and Analysis System (AKTA pure, GE, USA) with Superdex 200 10/300 GL (GE, USA) under 280 nm UV absorption. Morphological display was performed by TEM (Tecnai 12, Philips) with negative staining by 2% phosphotungstic acid. The absorption spectra of the ODP‐TH were measured by UV–vis spectrometry (UV‐2550, Shimadzu), and the fluorescence spectra were tested by fluorescence spectroscopy (Edinburgh Instruments, FS5 Spectrofluorometer) The detection methods of free sulfhydryl groups in different samples referred to a previous report. Oxygen release of ODP‐TH and HbO_2_ were determined by a dissolved oxygen meter (JPB‐607A, China), respectively.

### Cell Culture

4T1 and U87MG‐luc cells were obtained from the National Collection of Authenticated Cell Cultures (Shanghai, China) and cultured in DMEM mixed with 1% penicillin–streptomycin solution and 10% fetal bovine serum (FBS) at 37 °C with 5% CO_2_. The hypoxia cells were cultured with 5% O_2._

### Detection of Binding Affinity via Microscale Thermophoresis

TFRs were labeled with Monolith RED‐NHS protein labeling kit (NanoTemper Technologies GmbH) according to the kit instructions. TFRs protein labeling solution (5 n m ) was mixed with a PBS solution containing 0.05% tween and an initial concentration of TH nano shell (20 µ m ) in a ratio of 1:1. The mixture reacted at 25 °C for 5 min in the dark and was loaded into Premium Coated Capillaries (NanoTemper Technologies GmbH). All samples were tested by the Nanotemper Monolith NT.115 pico instrument.

### In Vitro Cytotoxicity Assays

First, the logarithmic phase cells were collected. Next, 100 µL cell suspension which contains 1000–10 000 cells was moved into each well of a 96‐well flat and incubated with 5% CO_2_ at 37.0 °C until it was fully filled with cells. Thereafter, culture medium was removed and gradient concentrations of samples were added into each well after being rinsed by PBS. After incubation for different times, the culture medium was changed and cells were irradiated for 10 min or kept in the dark continually. Subsequently, after the cells were cultured for different time, 10 µL MTT solution (5 mg/mL , i.e., 0.5% MTT) was added to each well for another 4 h. Finally, the culture medium was removed. Dimethyl sulfoxide (150 µL) was added to each well and shaken at a low speed for 10 min to make the crystals fully dissolved. The absorbance value of each sample was measured by enzyme‐labeled instrument (INFINITE 200 PRO, Switzerland). The viability of cell was calculated as follows:

(1) $$\mathrm{Cell}\;\mathrm{viability}\left(\%\right)=\frac{{\mathrm{OD}}_{\mathrm{positive}}\;-{\;\mathrm{OD}}_{\mathrm{experiment}}}{{\mathrm{OD}}_{\mathrm{positive}}\;-{\;\mathrm{OD}}_{\mathrm{control}}}\times 100\%$$

OD_positive_ is the absorbance of untreated cells and OD_control_ is the only culture medium.

### Establishment of BBB Model In Vitro

The bEnd.3 cells (1.6 × 10^5^ mL^−1^) were inoculated on the PET membrane (3 µm) of Transwell plate (12 pores) and cultured for 78 h.

### Detection of Reactive Oxygen Species In Vitro

The 4T1 cells were evenly seeded on the six well plate with a cover glass at the bottom for 12 h and incubated with PpIX (5 µg mL^−1^), P‐TH (5 µg mL^−1^ PpIX), and OP‐TH (5 µg mL^−1^ PpIX) for 4 h. Next, cells were treated with ROS detection kit for half an hour in dark and administered for 10 min with 650 nm laser (100 mW cm^−2^) after the medium was changed. Subsequently, all samples were washed three times with PBS and fixed with 4% paraformaldehyde for 15 min. Finally, cells were stained with DAPI for 20 min and then observed by CLSM. ROS (Ex/Em = 504/529 nm). The production of ROS was also detected by flow cytometry.

### In Vitro Cellular Uptake

The 4T1 cells were evenly seeded on the 12‐well plate with a cover glass at the bottom for 12 h at 37 °C. The cells were incubated with ODP‐TH (5 µg mL^−1^ PpIX) for various times. After being washed with PBS for at least three times, the treated cells were fixed with 4% paraformaldehyde for 20 min, and then DAPI staining were performed for another 15 min as required. All samples were observed by CLSM at the excitation wavelengths of 405 (emission bandpass: 610–660 nm) and 496 nm (emission bandpass: 550–590 nm).

### Analysis of Mitochondrial Membrane Potential

Mitochondrial membrane potential was assessed by JC‐1 Kit. The 4T1 cells were evenly seeded on the six well plate with a cover glass at the bottom for 12 h and then treated with PpIX (5 µg mL^−1^), P‐TH (5 µg mL^−1^ PpIX), and OP‐TH (5 µg mL^−1^ PpIX) for another 4 h. Cells in different groups were administrated for 10 min with 650 nm laser (100 mW cm^−2^) or not, then incubated with JC‐1 at 37 °C for 30 min. All samples were carried out by flow cytometry and CLSM. Monomer of JC‐1 (Ex/Em = 514/529 nm), J‐aggregates (Ex/Em = 585/590 nm).

### Detection of Cell Live/Dead Co‐Staining

The synergistic antitumor effect of ODP‐TH was measured by Calcein/PI cell viability assay kit. The 4T1 cells were evenly seeded on the six well plate with a cover glass at the bottom for 12 h. Thereafter, cells were incubated with PpIX + Dox (5 µg mL^−1^), DP‐TH (5 µg mL^−1^ PpIX and Dox), and ODP‐TH (5 µg mL^−1^ PpIX and Dox) for 4 h, respectively. Further, cells in different groups were lasered for 10 min (100 mW cm^−2^) or not and then cultured at 37 °C for another 12 h. All samples were co‐stained by Calcein‐AM/PI followed with the manufacturer's instruction and recorded by CLSM, respectively. Calcein‐AM (Ex/Em = 490/515 nm), PI (Ex/Em = 545/617 nm).

### WB Assay of Hif‐1*α* and P‐gp

4T1 cells were seeded in cell culture bottle at 37 °C for 48 h under normoxia or hypoxia conditions. Hypoxia cells were treated with ODP‐TH (5 µg mL^−1^ PpPX and Dox) and cultured at 37 °C for 1, 4, and 12 h, respectively. After that, all cells were collected and added to RIPA lysis buffer containing protein inhibitor cocktail (Thermofisher, USA) for 30 min. The concentration of total protein was measured by the BCA protein assay kit. Then the same amount of protein was electrophoresed in 10% SDS‐PAGE gel at 80 V for 0.5 h then increased to 120 V for another 75 min. Thereafter, the protein was transferred onto PVDF membrane pretreated with methanol for 2 min. The membrane was sealed with 5% skim milk and incubated overnight with anit‐Hif‐1*α* and anti‐P‐gp antibody at 4 °C. After being washed by 0.1% TBST for at least 3 times, the membrane was incubated with goat anti‐mouse secondary antibody diluted with 5% skim milk at 37 °C for 2 h. Finally, after being washed by 0.1% TBST for three times, the membrane was visualized with chemiluminescence imaging system (Tanon‐5200, China).

### RT‐qPCR Analysis of Expression Level of HIF‐1*α* and MDR1

RT‐qPCR was applied to semi‐quantitatively evaluate the expression levels of Hif‐1*α* and MDR1. The method of cell culture and pretreatment were similar to that of WB. A group of anoxic cells were incubated with ODP‐TH (5 µg mL^−1^ PpIX and Dox) for 2 h. Total RNA was extracted from 4T1 cells with Trizol reagent (Sigma, USA), and then reversely transcripted into cDNA with cDNA RT kit (Toyobo, Japan) followed with the instructions from a manufacturer. Then, the RT‐qPCR was carried out by Thunder Bird SYBR qPCR mix (Toyobo, Japan). GAPDH was selected as an endogenous reference. The primers gene sequence of HIF‐1*α* were 5″‐AGCAATTCTCCAAGCCCTCC (Forward) and CGTAACTGGTCAGCTGTGGT‐3″ (Reverse). The primers gene sequence of MDR1 were 5″‐AGTGGCTCTTGAAGCCGTAA (Forward) and AACACCAGCAT‐CAAGAGGGG‐3″ (Reverse). The primers gene sequence of GAPDH were 5″‐CTACTCGCGGCTTTACGGG (Forward) and CTCGCTCCTGGAAGATGGTG‐3″ (Reverse).

### Animal Model Establishment

All SPF female BALB/c nu mice (8 weeks, 19 g) were obtained from Hangzhou ZiYuan Laboratory Animal Technology Co., Ltd. All the animal related experiments were conducted in accordance with the requirements of the Institutional Animal Care and Use Committee (IACUC) of Nanjing University and Nanjing Medical University, the IACUC number are 2104016 and 2205021, respectively: 1) Mice were randomly divided into 13 groups and each group comprised 5 mice. 200 µL of PBS containing 4T1 cells (5 × 10^5^) were injected into the right hind limb of mice. When the average tumor volume reached 100 mm^3^ at 1 week post‐injection before further experiments could be performed; and 2) before implantation, 85–90% confluent U87MG‐luc cells were trypsinized, rinsed with DMEM+10% fetal bovine serum (FBS), and centrifuged at 1000 rpm for 4 min. The cell pellet was resuspended in DMEM and placed on ice. The concentration of viable cells was adjusted to 6 × 106 cells mL^−^ of DMEM. Each BALB/c nude (6 weeks old) mouse was anesthetized with a mixture of oxygen and isoflurane (induction: 5%; maintenance: 2%) and placed in the scanner, where the isoflurane‐oxygen mixture was administered through a nose cone fixed onto the animal holder and placed in a stereotactic frame (Sansbio, Nanjing, China). After shaving and disinfection of the skin, a sagittal incision was made to expose the skull, followed by a burr hole 1 mm anterior and 2 mm lateral relative to the bregma using a small drill. Cell suspension was injected into a depth of 3 mm from the skull surface, using a 10 µL Hamilton syringe (Reno, NV, USA) with a 26s‐gauge needle mounted on a stereotactic holder. On completion of the injection, the needle was left in place for 1 min and withdrawn slowly. The scalp incision was then closed with surgical sutures. The animals were injected intramuscularly with 0.1 mL/mouse of 80 U mL^−1^ benzylpenicillin sodium solutions for prevention of infection and returned to their home cages.

### In Vivo Imaging Analysis

IVIS Lumina XR (PerkinElmer, USA) was carried out for in vivo FLI and BLI imaging of 4T1 and U87MG tumor bearing BALB/c nu mice. OD‐TH (2.5 mg kg^−1^ Dox) was i.v. injected into mice when the tumor grew to about 200 mm^3^. After isoflurane anesthesia, the images were recorded from 1 to 48 h (Ex = 500 nm/Em reference DsRed). The solid tumor and major organs were excised 48 h later for FLI. In semi‐quantitative FLI analysis, DOX (2.5 mg kg^−1^) and OD‐TH (2.5 mg kg^−1^ Dox) were i.v. injected into different 4T1 tumor bearing mice, respectively. 24 later, their solid tumors and main organs were obtained. PBS solution (10 µL g^−1^) D‐fluorescein potassium salt (15 mg mL^−1^) was i.p. injected into U87MG tumor bearing BALB/c nu mice. The BLI of the mice head was measured 12 min post injection. The same region‐of‐interest (ROI) was circled by Living Image software (PerkinElmer, USA) after Flat Field Correction and Cosmic Ray Corrections to compare the FL intensity of parallel parts.

### Detection of Singlet Oxygen In Vivo

The tumor bearing mice were intravenous injected with PpIX (5 mg kg^−1^), P‐TH (5 mg kg^−1^ PpIX), and OP‐TH (5 mg kg^−1^ PpIX) respectively. The mice in each group were intraperitoneally injected with SOSG 4 h later, and then irradiated for 10 min with 650 nm laser (100 mW cm^−2^). The mice in each group were killed to collect solid tumors after 6 h administration, and the frozen sections (7 µm) of tumor tissue were dyed with DAPI for 20 min. At last, the samples were washed with PBS and observed by CLSM. SOSG (Ex/Em = 488/525 nm).

### Immunofluorescence Detection of Hypoxia Tissues

Immunofluorescence assay of HIF‐1*α* and hypoxia marker was performed. 4T1 tumor bearing mice with tumor volume of about 200 mm^3^ were selected and i.v. injected with PpIX, P‐TH, and OP‐TH (2.5 mg kg^−1^ PpIX), respectively. The tumor was dissected surgically 12 h post‐injection. After being embedded in an optimal cutting temperature compound (OCT), tumors were cut into tissue sections (7 µm) on a freezing microtome and attached to the slide. All tissues were incubated with primary anti‐Hif‐1*α* antibody for 2 h, and washed thrice with PBS. All samples were washed thrice with PBS containing 0.1% tween‐20 after being treated with secondary Alexa Fluor488‐conjugated goat anti‐rabbit IgG H&L antibody for 1 h. Finally, all samples were dyed with DAPI and recorded by CLSM. In the immunofluorescence study of hypoxia marker, the pretreatment of mice was similar to the above method. 6 h after the i.p. injection of pimonidazole HCl (Hypoxyprobe‐1 plus kit, Hypoxyprobe Inc., USA 60 mg kg^−1^), the tumors were dissected and made into frozen tissue sections (7 µm). First, tumor tissues were treated with anti‐pimonidazole antibody (dilution rate 1:200, Hypoxyprobe‐1 plus kit). Next, horseradish peroxidase linked to goat anti‐mouse secondary antibody (dilution rate 1:200) was added according to the manufacturer's instructions. At last, all samples were stained with DAPI and observed under CLSM.

### Studies of Drug Distribution In Vivo

The distribution of drugs in vivo was evaluated by the FL intensity of Dox and PpIX in solid tumors and major organs. ODP‐TH (5 µg mL^−1^PpIX and Dox) was i.v. injected when the tumor volume of 4T1 bearing mice reached about 200 mm^3^. The tumor and main organs were obtained at 48 h post‐injection. Tumors and organs of each group were ground by tissue grinder (Tissuelyser‐GXF). After been centrifugated for 15 mins at the speed of 10K rounds per minute, the supernatant was taken out and repeated 3 times. The supernatants were merged and stood overnight after being mixed with 200 µL anhydrous methanol. After being centrifugated (10K rpm, 15 min) for 3 times, the supernatant was measured.

### Oxygen Partial Pressure Test In Vivo

The 4T1 tumor bearing mice were anesthetized with pentobarbital (1%, 80 mg kg^−1^) via intraperitoneal injection and fixed on the operating plate. The optical fiber sensors (Oxford Optronix, UK) were inserted into the liver, kidney, and tumor at different times to record the corresponding pO_2_ after intravenous injection. All pO_2_ were recorded after being stable for 1 min.

### Pharmacokinetics Analysis

The 4T1 tumor bearing mice were intravenously injected with Dox (10 mg kg^−1^) and ODP‐TH (36 mg kg^−1^). Venous blood (30 µL) was collected from the submaxillary vein of each mouse 0.25, 0.5, 1, 2, 4, 6, 12, and 24 h after injection. The separated serum was diluted 50 times with UP water. Mobile phase ratio: 6 (methanol):4 (UP water).

### Antitumor Efficiency in 4T1 Model

The mice were randomly divided into 13 groups when the average volume of tumor reached 100 mm^3^: 1) PBS; 2) PpIX + Laser; 3) Dox; 4) PpIX + Dox + Laser; 5) OP‐TH; 6) OP‐TH + Laser; 7) P‐TH + Laser; 8) D‐TH; 9) OD‐TH; 10) DP‐TH; 11) DP‐TH + laser; 12) ODP‐TH; and 13) ODP‐TH + laser. All mice were i.v. injected (5 mg kg^−1^) once every two days, and the groups (2, 4, 6, 7, 11, and 13) were irradiated with 650 nm laser for 10 min at 12 h post‐injection. The body weight and tumor volume (volume = length × width^2^ × 0.5) were recorded before each administration. Four representative groups were selected and their morphological changes were recorded every three days. On the 15th day after the first administration, the major organs and tumors of mice were dissected and the weight of solid tumors was recorded. All samples were stained with H&E for histological analysis.

### Antitumor Efficiency in Orthotopic Glioma Model

The U87MG‐luc bearing mice were randomly divided into 6 groups after 10 days of tumor cell inoculation, there were 3 mice in each group. All mice were administered and imaged in vivo every three days. Body weight was recorded every three days. All mice were sacrificed on D15. Brain, liver, heart, spleen, and kidney were collected for pathological research.

### Physiological Index Detection In Vivo

Blood samples were collected from mice with 15 days ODP‐TH + Laser administration. All tests were conducted according to the kit manufacturer's instructions.

### Statistical Analysis

All data were presented as mean ± standard deviation. The sample quantity (*n*) for each test is indicated in the legend. The statistical significance was determined using a two‐tailed unpaired *t*‐test by SPSS 24, for multiple comparisons, the data were analyzed by a one‐way ANOVA. The levels of significant differences were indicated by * (*p < 0.05, **p < 0.01, ***p < 0.001).The analytical graphs were produced by GraphPad Prism 9.

## Conflict of Interest

The authors declare no conflict of interest.

## Supporting information

Supporting Information

## Data Availability

The data that support the findings of this study are available from the corresponding author upon reasonable request.

## References

[1] a) J. D. Martin , G. Seano , R. K. Jain , Annu. Rev. Physiol. 2019, 81, 505; 30742782 10.1146/annurev-physiol-020518-114700 PMC6571025

[2] a) F. Li , Y. Du , J. Liu , H. Sun , J. Wang , R. Li , D. Kim , T. Hyeon , D. Ling , Adv. Mater. 2018, 30, 1802808; 10.1002/adma.201802808 29999559

[3] a) B. W. Henderson , T. J. Dougherty , Photochem. Photobiol. 2010, 55, 145; 10.1111/j.1751-1097.1992.tb04222.x 1603846

[4] a) Z. Liu , T. Cao , Y. Xue , M. Li , W. Zhang , Angew. Chem., Int. Ed. 2020, 59, 3711; 10.1002/anie.201914434 PMC7028480 31808983

[5] a) Z. Ge , L. Guo , G. Wu , J. Li , Q. Li , Small 2020, 16, 1904857; 10.1002/smll.201904857 32191376

[6] a) M. Heuvel‐Eibrink , E. Wiemer , M. Boevere , B. Holt , P. Sonneveld , Blood 2001, 97, 3605; 11369657 10.1182/blood.v97.11.3605

[7] a) Y. Du , C. Yang , F. Li , H. Liao , Z. Chen , P. Lin , N. Wang , Y. Zhou , J. Y. Lee , Q. Ding , Small 2020, 16, 2002537; 10.1002/smll.202002537 32519453

[8] a) L. Duan , X. Yan , A. Wang , Y. Jia , J. Li , ACS Nano 2012, 6, 6897; 22732258 10.1021/nn301735u

[9] Z. Luo , H. Tian , L. Liu , Z. Chen , R. Liang , Z. Chen , Z. Wu , A. Ma , M. Zheng , L. Cai , Theranostics 2018, 8, 3584. 30026868 10.7150/thno.25409 PMC6037038

[10] a) T. Hao , Z. Luo , L. Liu , M. Zheng , Z. Chen , A. Ma , R. Liang , Z. Han , C. Lu , L. Cai , Adv. Funct. Mater. 2017, 27, 1603856;

[11] a) H. F. Wang , R. Ran , Y. Liu , Y. Hui , B. Zeng , D. Chen , D. A. Weitz , C. X. Zhao , ACS Nano 2018, 12, 11600; 30380832 10.1021/acsnano.8b06846

[12] a) L. Rao , Q. F. Meng , Q. Huang , Z. Wang , G. T. Yu , A. Li , W. Ma , N. Zhang , S. S. Guo , X. Z. Zhao , Adv. Funct. Mater. 2018, 28, 1803531;

[13] a) M. Cazzola , H. A. Huebers , M. H. Sayers , A. P. Macphail , C. A. Finch , Blood 1985, 66, 935; 2994783

[14] a) S. B. Ruan , L. Qin , W. Xiao , C. Hu , Y. Zhou , R. R. Wang , X. Sun , W. Q. Yu , Q. He , H. L. Gao , Adv. Funct. Mater. 2018, 28, 1802227;

[15] S. Z. Ren , B. Wang , X. H. Zhu , D. Zhu , M. Liu , S. K. Li , Y. S. Yang , Z. C. Wang , H. L. Zhu , ACS Appl. Mater. Interfaces 2020, 12, 24662. 32394704 10.1021/acsami.0c08534

[16] C. K. Riener , G. Kada , H. J. Gruber , Anal. Bioanal. Chem. 2002, 373, 266. 12110978 10.1007/s00216-002-1347-2

[17] M. N. Prout , M. Richards , K. J. Chung , P. Joo , H. L. Davis , Cancer 2015, 39, 62. 10.1002/1097-0142(197701)39:1<62::aid-cncr2820390112>3.0.co;2-j 188540

