# Supplementary material for: Multifunctional Protein Hybrid Nanoplatform for Synergetic Photodynamic‐Chemotherapy of Malignant Carcinoma by Homologous Targeting Combined with Oxygen Transport
Source: Adv Sci (Weinh). 2022 Dec 21;10(5):2203742. doi: 10.1002/advs.202203742 (PMC9929260; doi:10.1002/advs.202203742)
Supplement: Supplementary file 1 — Supporting Information [file ADVS-10-2203742-s001.pdf]

ADVANCED SCIENCE
Open Access

Supporting Information

for *Adv. Sci.*, DOI 10.1002/advs.202203742

Multifunctional Protein Hybrid Nanoplatform for Synergetic Photodynamic-Chemotherapy of Malignant Carcinoma by Homologous Targeting Combined with Oxygen Transport

Song-Yu Wu, Ya-Xi Ye, Qing Zhang, Qian-Jin Kang, Zhu-Min Xu, Shen-Zhen Ren, Fan Lin, Yong-Tao Duan, Hao-Jun Xu, Zi-Yi Hu, Sui-Sui Yang, Hai-Liang Zhu*, Mei-Juan Zou* and Zhong-Chang Wang**

Supporting Information

A Multifunctional Nanoplatform Based on Protein Hybrid for Enhancing Synergetic Photodynamic-Chemo Therapy Effect by Homologous Targeting Combined with Oxygen Transport

Song-Yu Wu[†,a], Ya-Xi Ye[e], Qing Zhang[a], Qian-Jin Kang[a], Zhu-Min Xu[a], Shen-Zhen Ren[d], Yong-Tao Duan*[,c], Fan Lin[b], Haojun Xu[b], Ziyi Hu[b], Suisui Yang[b], Hai-Liang Zhu*[, a, c], Meijuan Zou*[, b], Zhong-Chang Wang*[,a]

[a]*State Key Laboratory of Pharmaceutical Biotechnology, School of Life Sciences, Institute of Artificial Intelligence Biomedicine, Engineering Research Center of Protein and Peptide Medicine，Ministry of Education，Nanjing University, Nanjing 210023, PR China*

[b]*Department of Pharmacology, School of Basic Medical Sciences, Nanjing Medical University, 101 Longmian Avenue, Nanjing, Jiangning, 211166, China*

[c]*Henan provincial key laboratory of children's genetics and metabolic diseases，Children's Hospital Affiliated to Zhengzhou University, Zhengzhou University, Zhengzhou 450018, China*

[d]*Key Laboratory of Molecular Biophysics Hebei Province, Institute of Biophysics School of Sciences, Hebei University of Technology, Tianjin, 300401, China*

[e]*Institute of Pharmaceutical Biotechnology, School of Biology and Food Engineering, Suzhou University, Suzhou 234000, PR China*

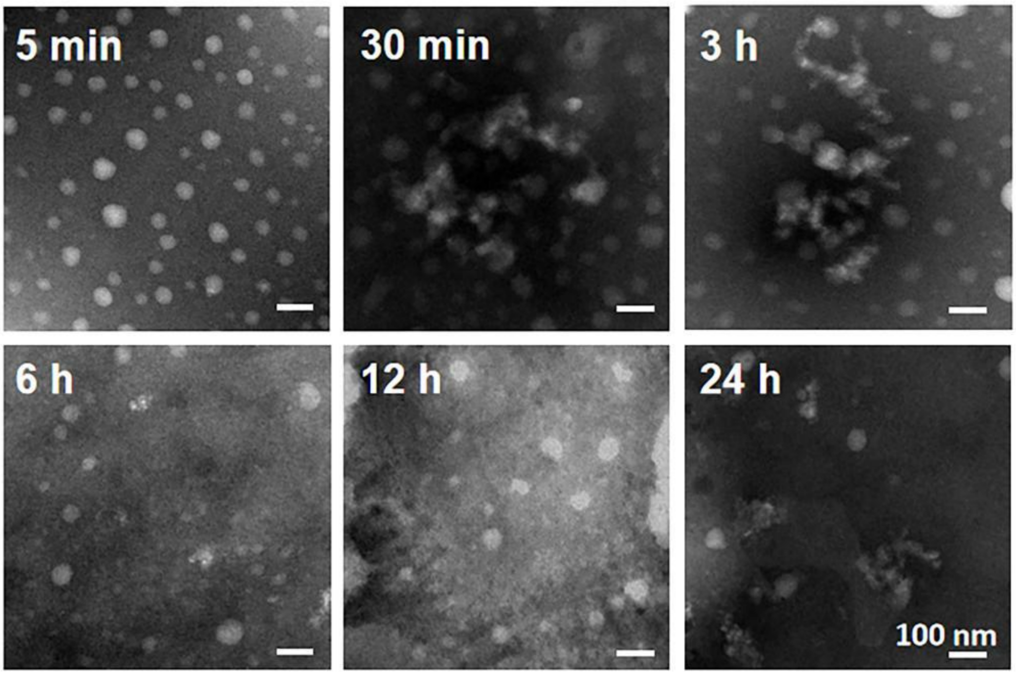

Figure S1. TEM of ODP-TH incubated with GSH (5 mM) in PBS at 25 ℃ from 5 min to 24 h. Scale bar = 100 μm.

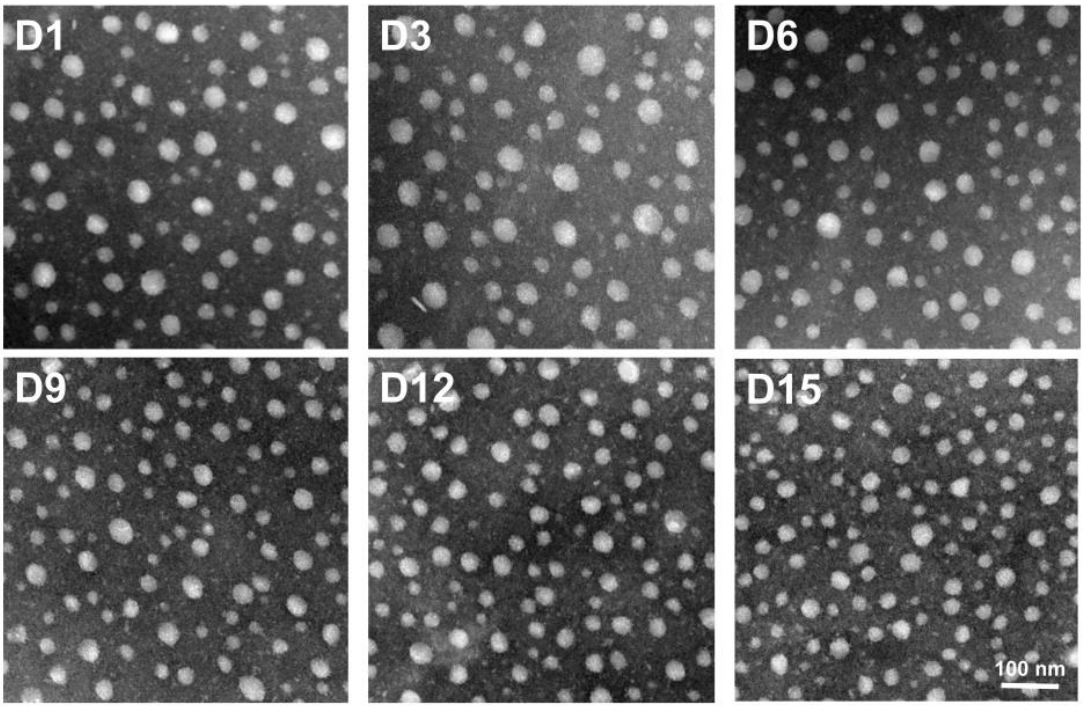

Figure S2. The TEM images of ODP-TH dispersed in PBS on D1, D3, D6, D9, D12, D15 at 25 ℃. Scale bar = 100 μm.

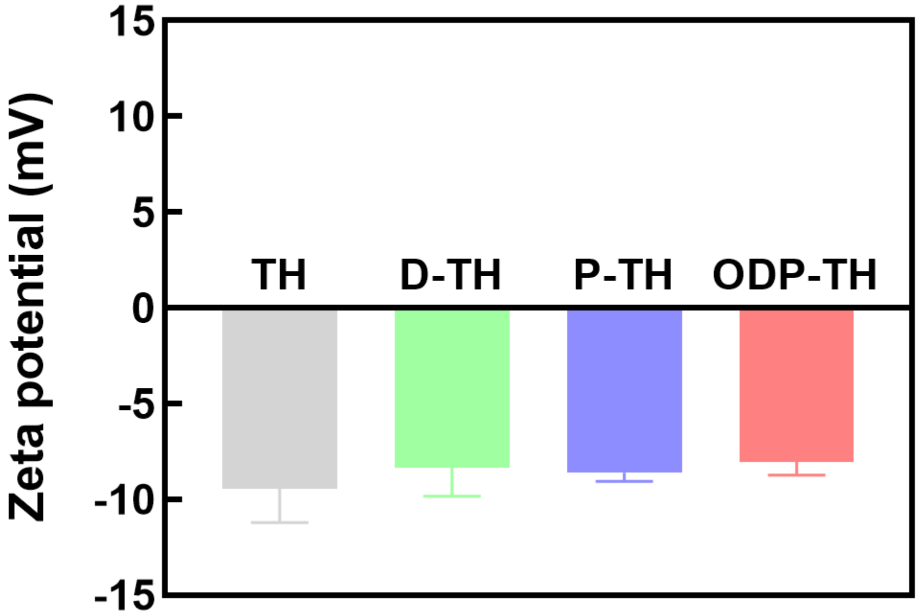

Figure S3. Zeta poteintial of TH nanoshell, single loaded D-TH and P-TH, dual loaded ODP-TH. Mean ± SD, n = 3.

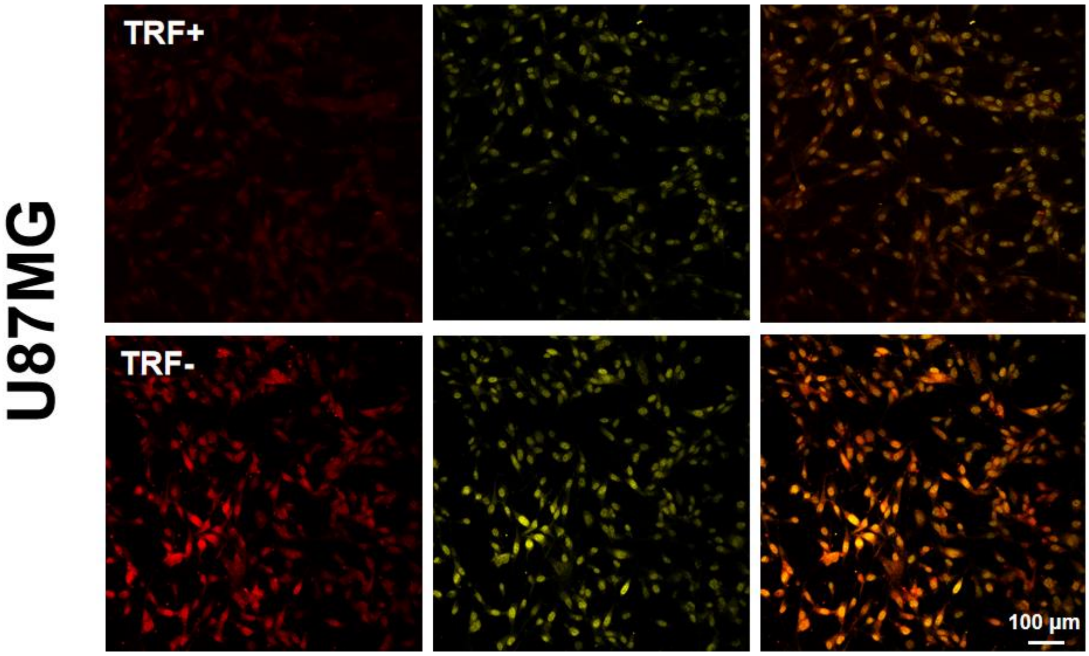

Figure S4. CLSM images of ODP-TH uptake in U87MG cells treated with TRF (500 μM) for 4 h and unpretreated U87MG cells. Scale bar = 100 μm

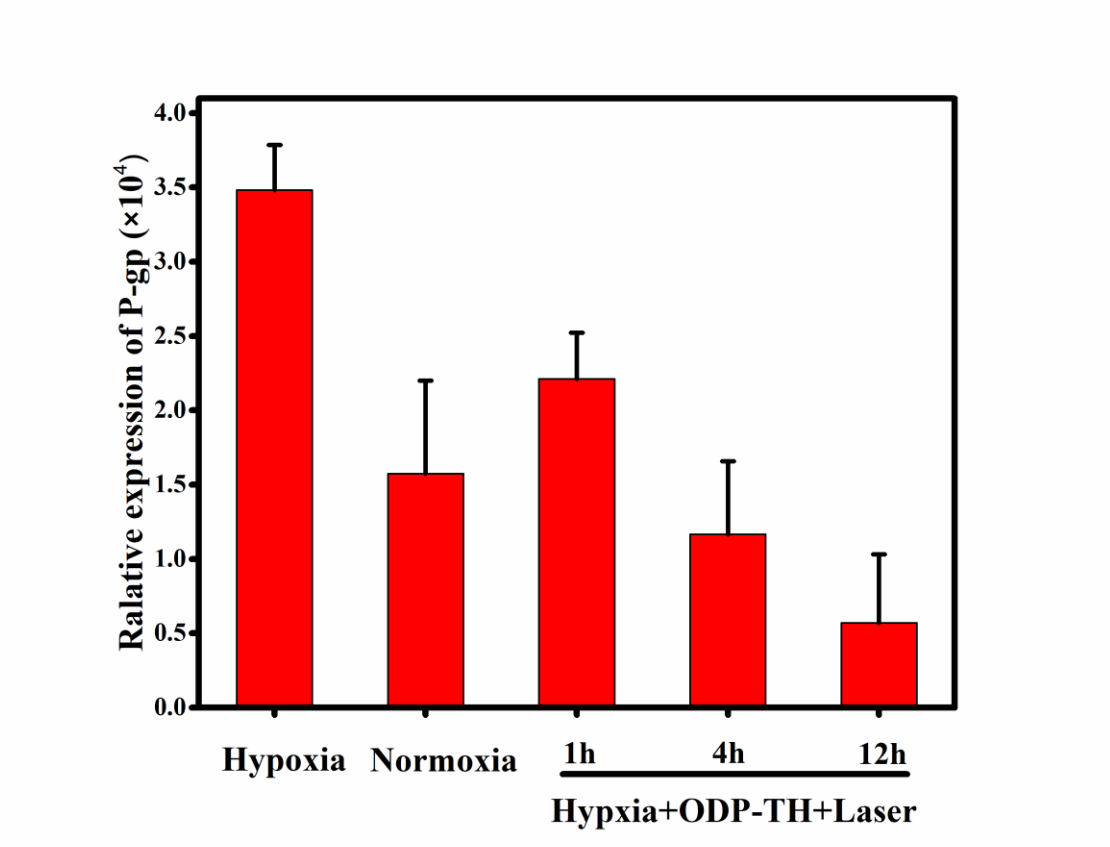

Figure S5. Semi-quantitative analysis of P-gp expression of 4T1 cells in different state. Mean ± SD, n = 3.

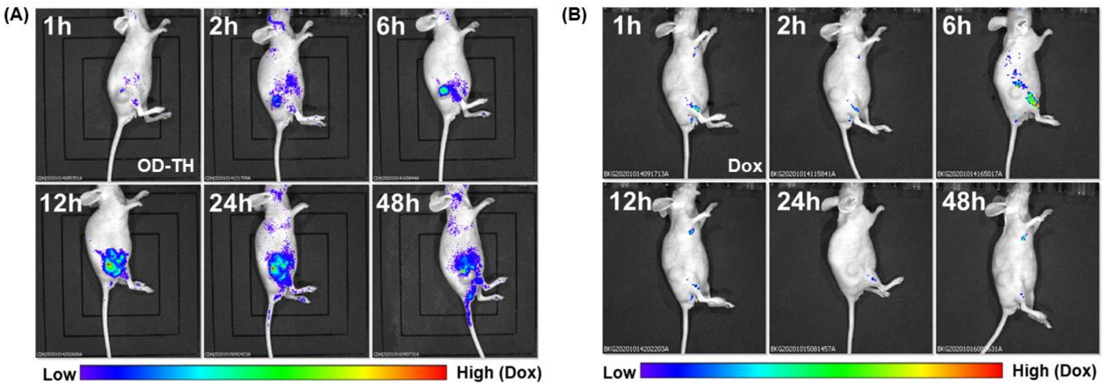

Figure S6. Time lapse fluorescence image of 4T1 tumor bearing BALB/c nu mice after injection of OD-TH (A) and Dox (B).

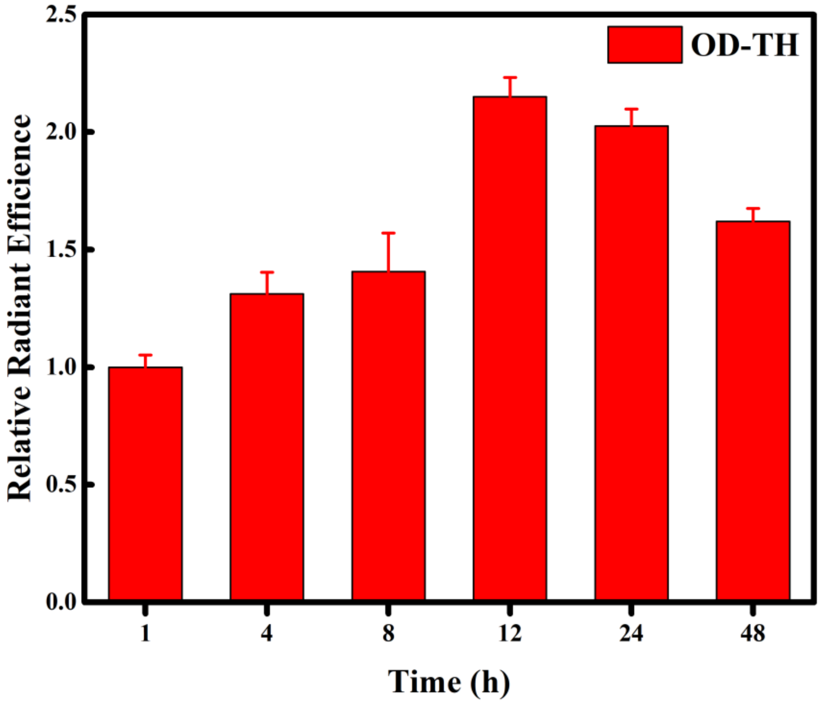

Figure S7. Semi-quantitative analysis of radiant efficience in ODP-TH treated mice tumour region at different time. All data were analyzed by software (imageJ). Mean ± SD, n = 3.

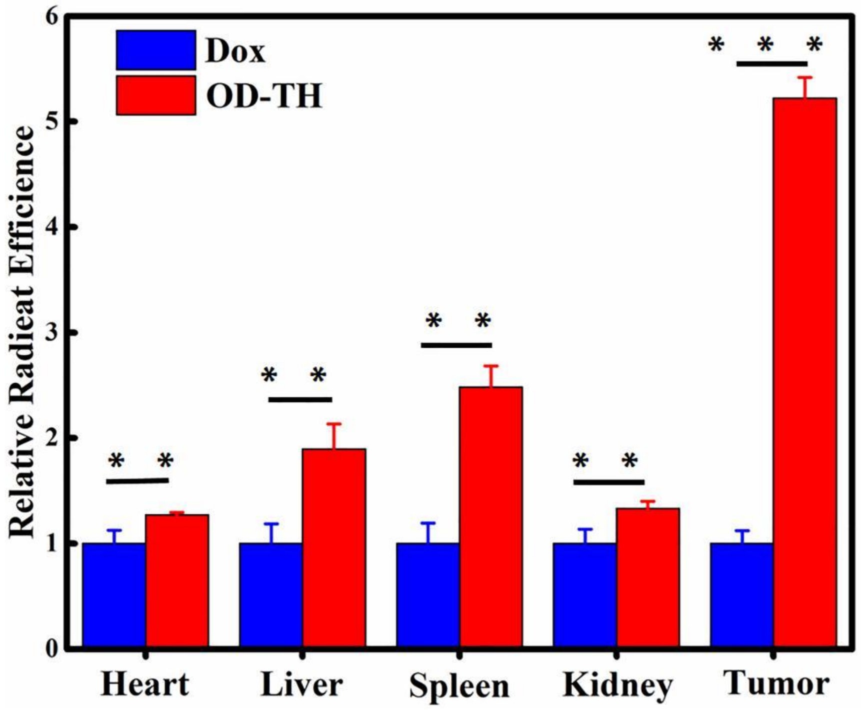

Figure S8. Relative fluorescence histogram of major organs and tumor 48 h after tail vein injection of Dox and OD-TH. Mean ± SD, n = 3. *P*-value were indicated by ** ($p < 0.01$) and *** ($p < 0.001$).

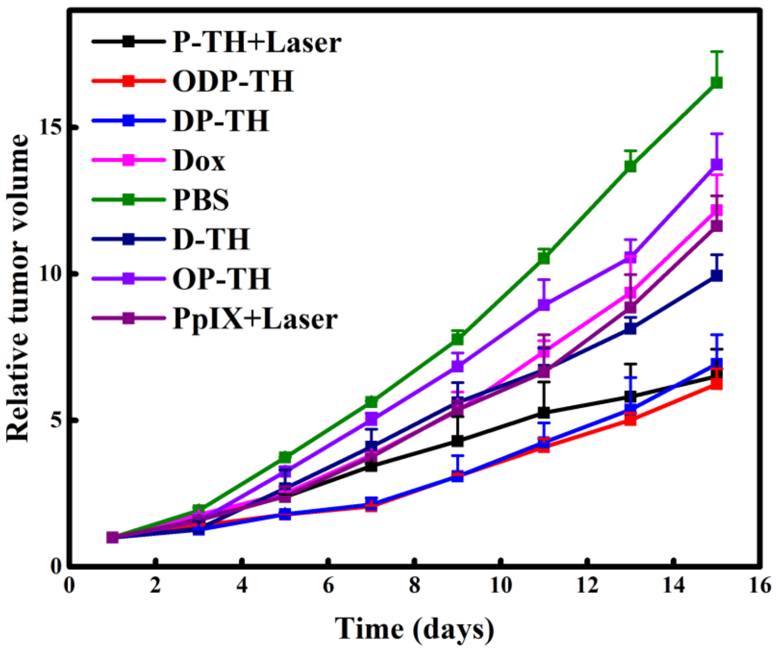

Figure S9. 4T1 tumor volume changes curves of different groups within 15 days after administration. Mean ± SD, n = 5.

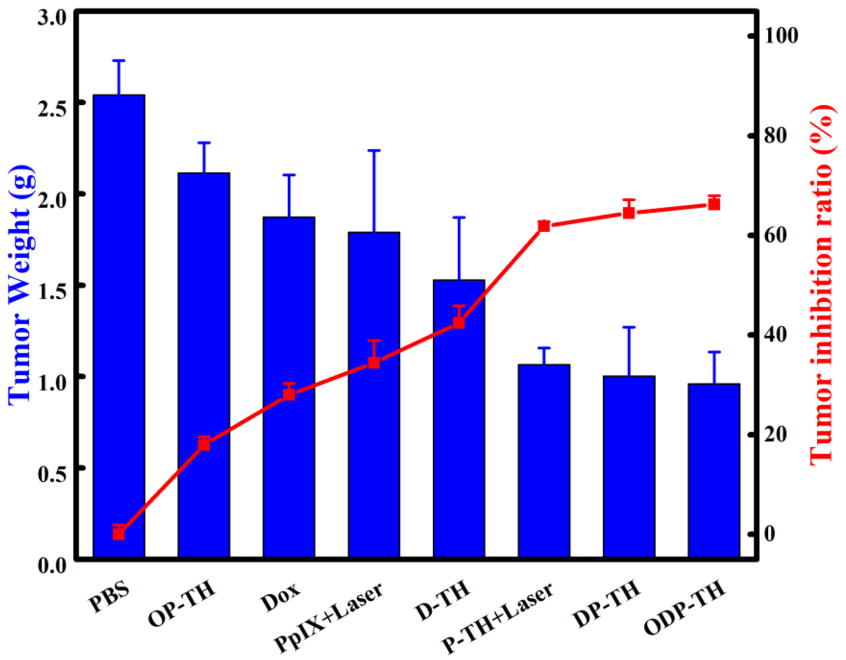

Figure S10. Tumor weight and inhibition rate in mice after 15 days after treatment. Mean ± SD, n = 5.

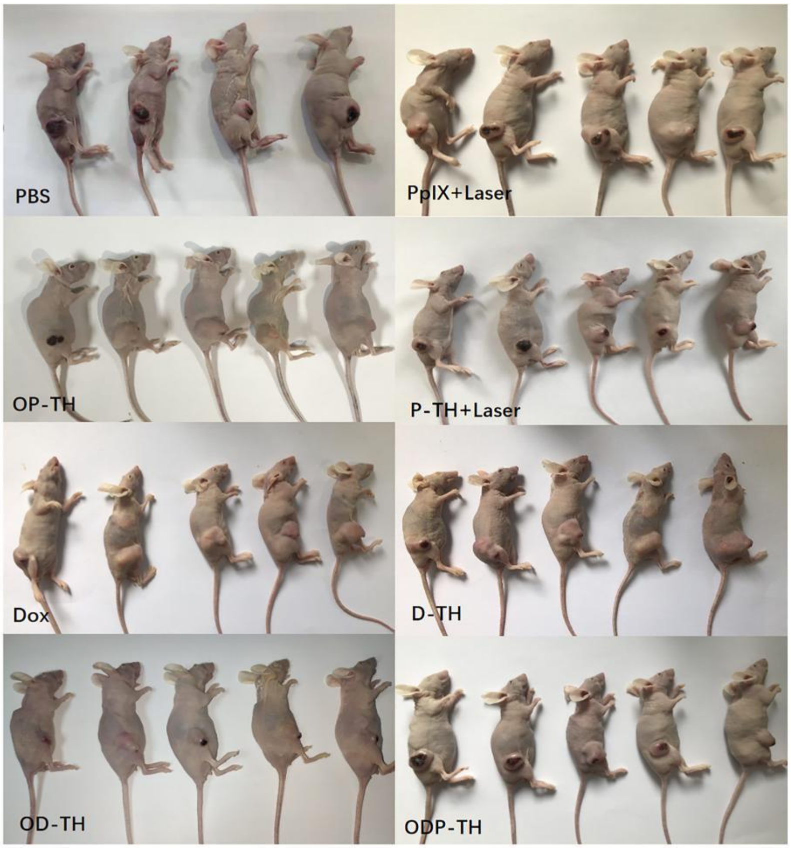

Figure S11. Pictures of mice at termination of treatment in eight groups.

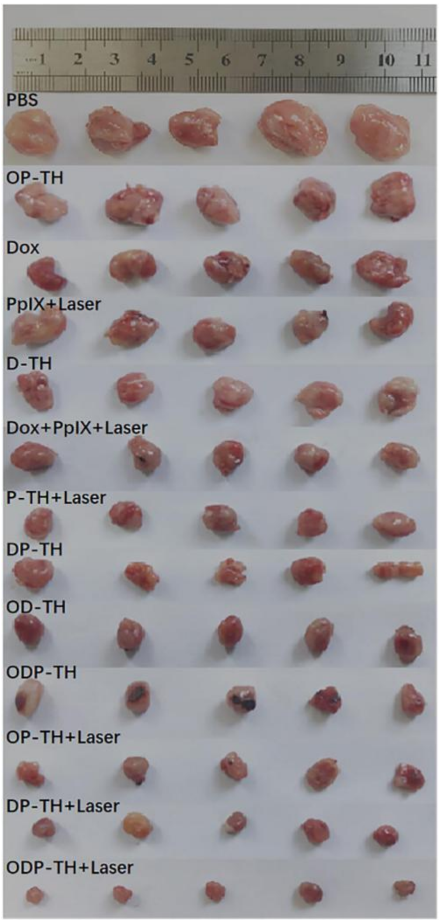

Figure S12. Images of all mice at the termination of treatment and tumor dissection.

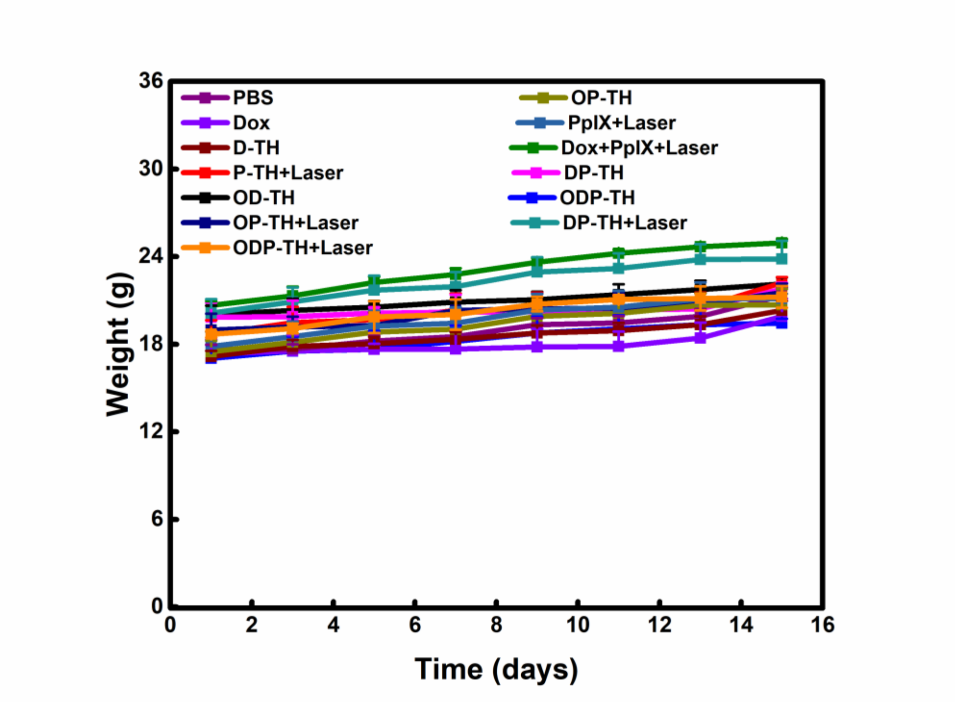

Figure S13. Weight change curves of all mice during treatment. Mean ± SD, n = 5.

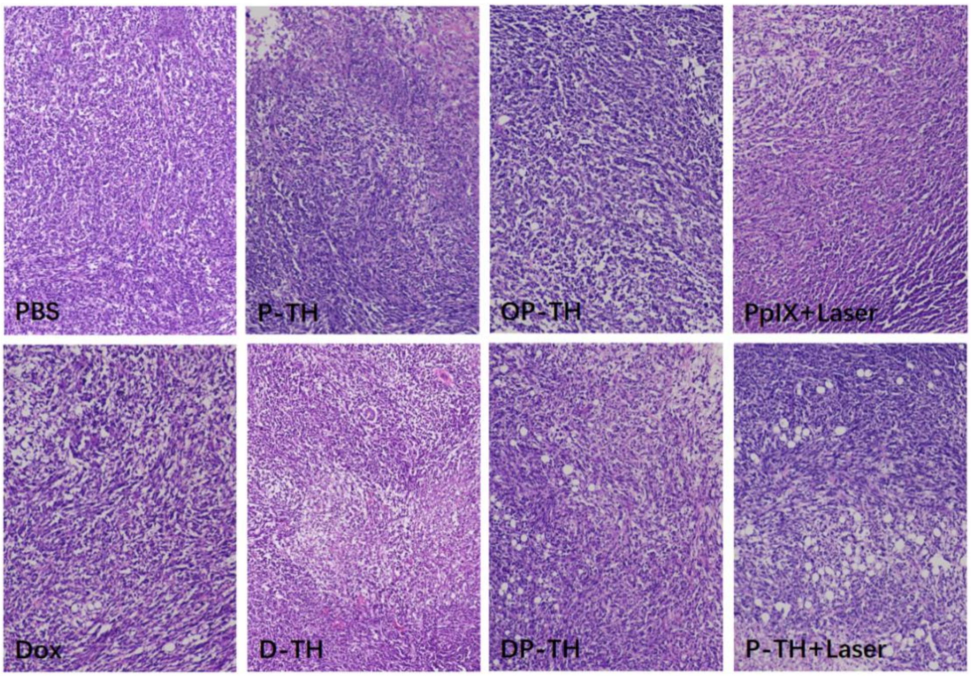

Figure S14. H&E staining images for the dissected tumors in seven groups. (100x)

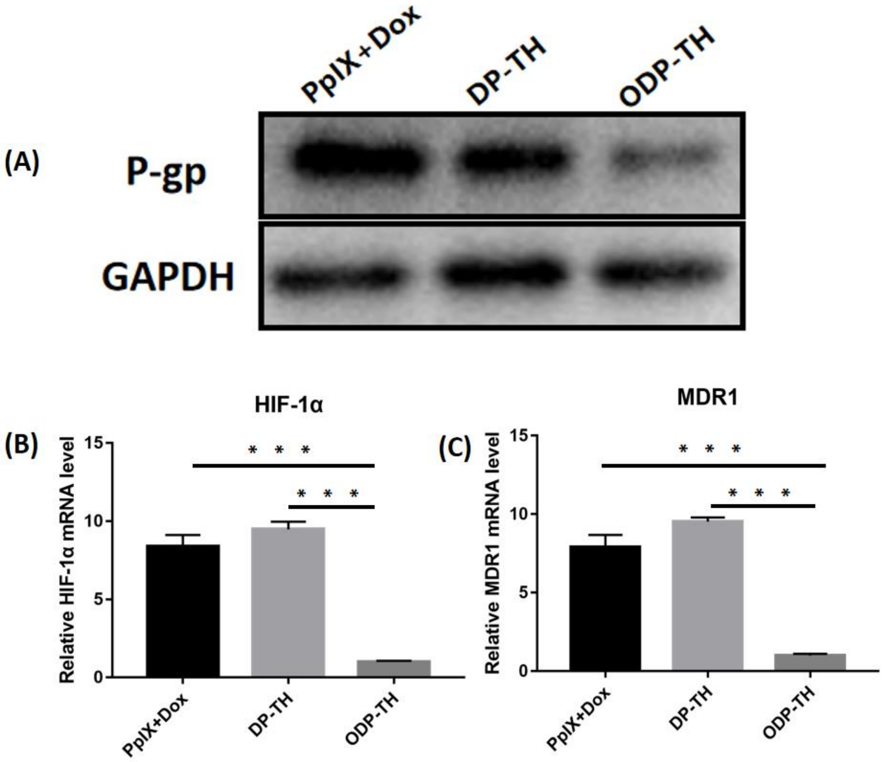

Figure S15. WB and qRT-PCR analysis of P-gp (A), HIF-1α (B) and MDR1 (C) expression of 4T1 tumor tissues treated with PpIX + Dox, DP-TH and ODP-TH for 12 h. Mean ± SD, n = 3. *P*-value were indicated by *** ($p < 0.001$).

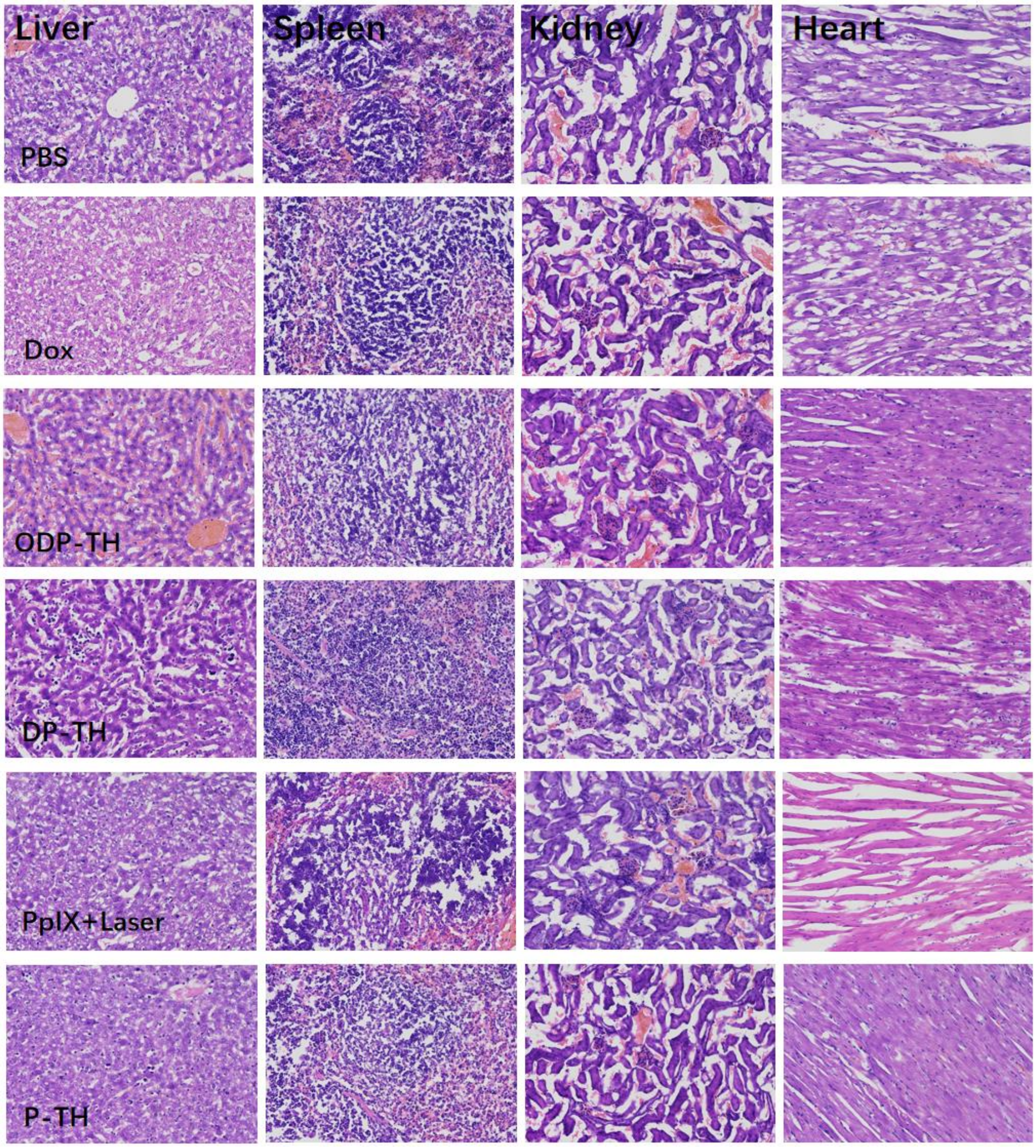

Liver
Spleen
Kidney
Heart
PBS
Dox
ODP-TH
DP-TH
PpIX+Laser
P-TH

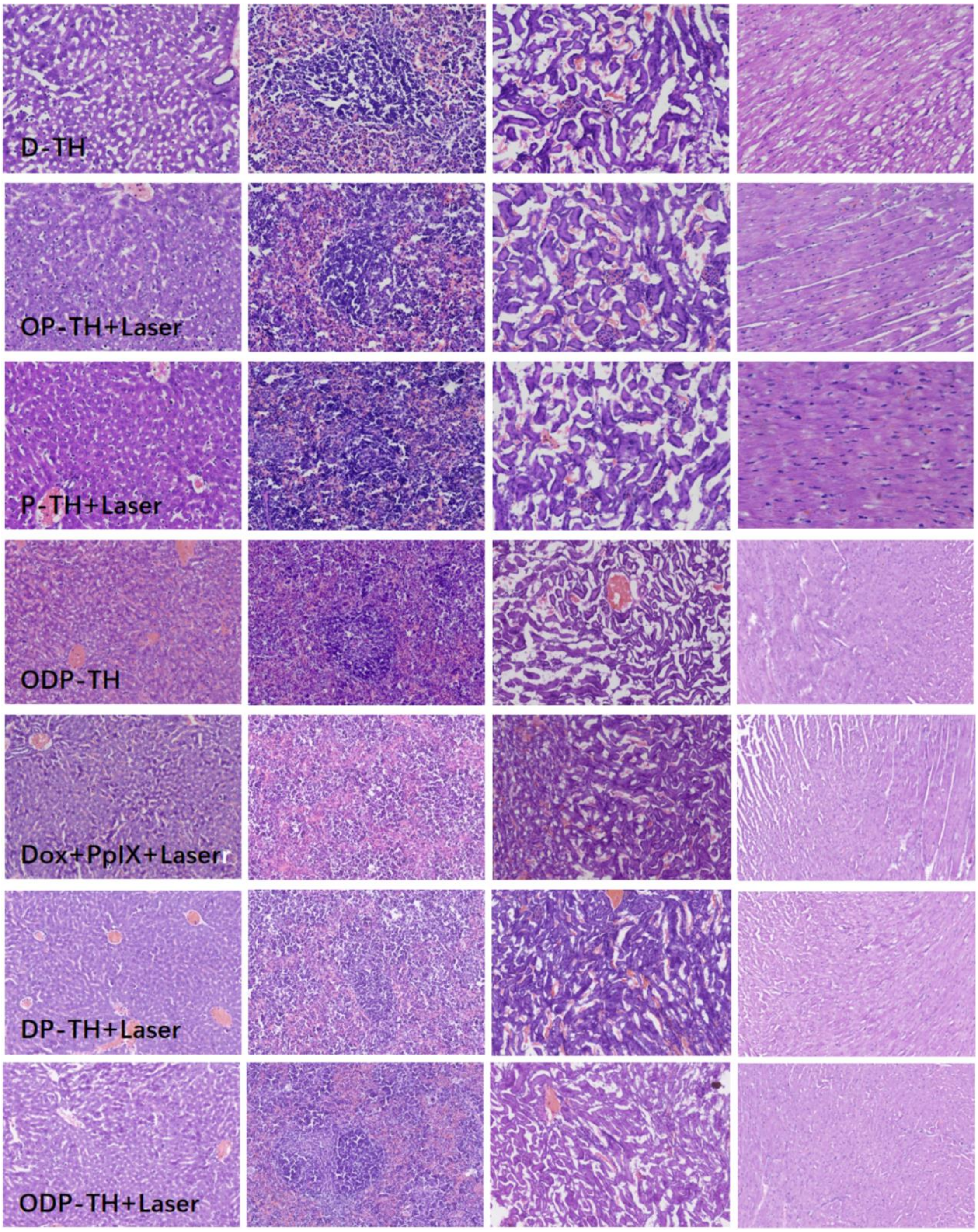

Figure S16. H&E staining images for the dissected major organs in 4T1 cells bearing mice. (200x)

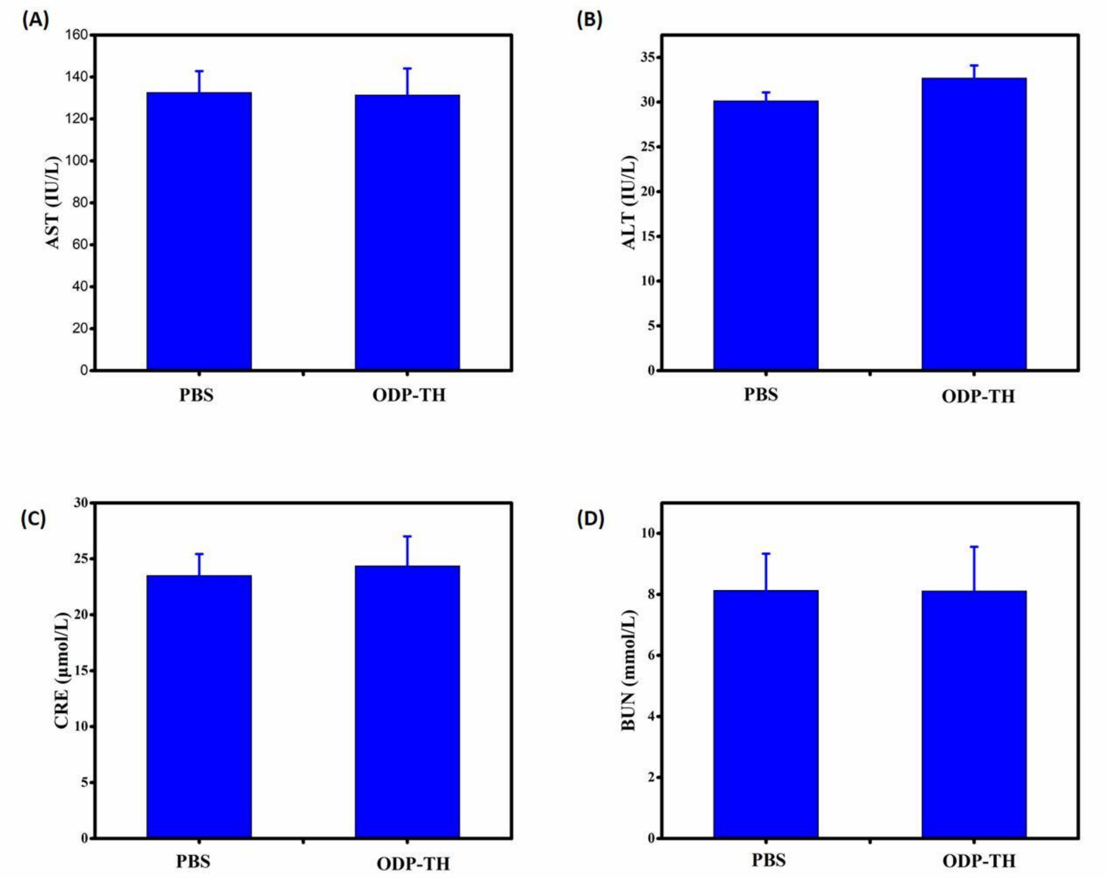

Figure S17. Detection of physiological indexes in blood of ODP-TH administrated mice. Mean ± SD, n = 3.

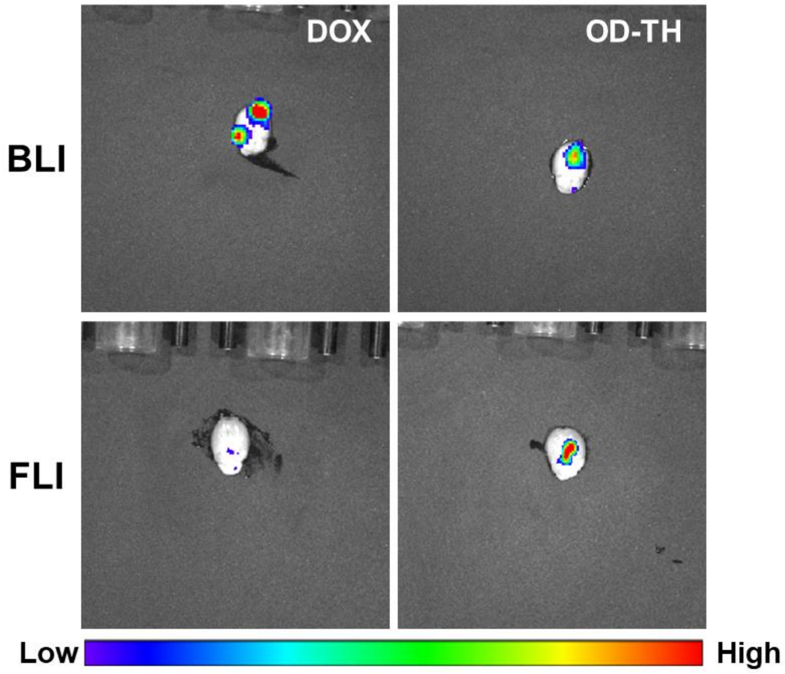

Figure S18. Imaging of bioluminescence (BLI) and fluorescence (FLI) co-localization in isolated brain tissue after 48 h treatment.

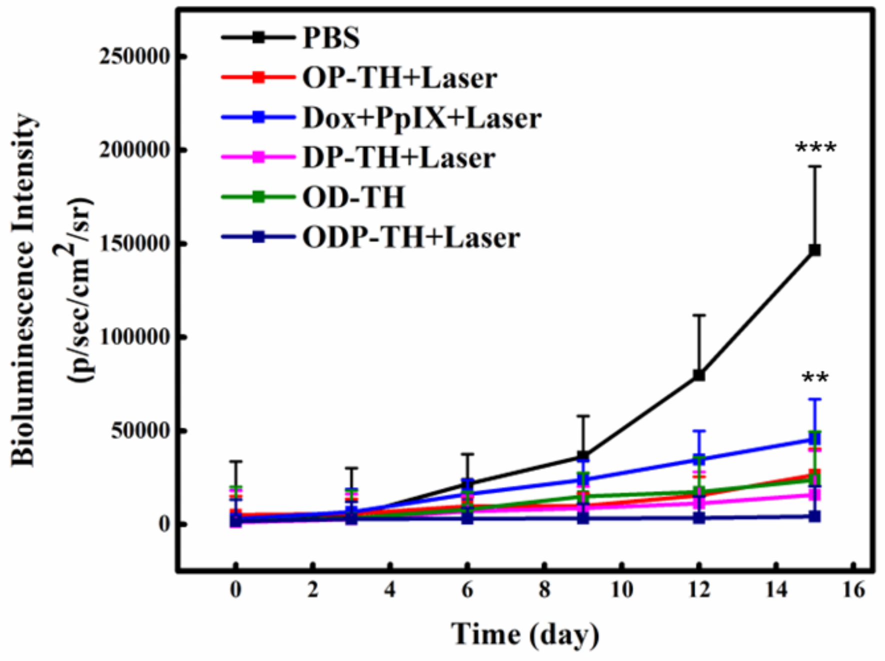

Figure S19. The brain BLI change curve of U87MG orthotopic transplanted mice. Mean ± SD, n = 3.

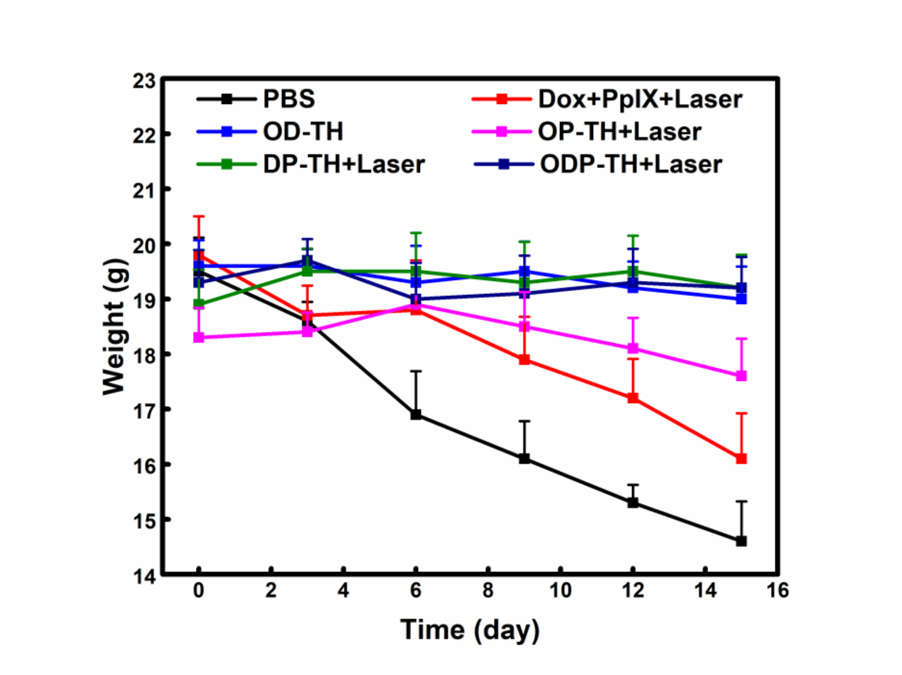

Figure S20. The weight change curve of U87MG orthotopic transplanted mice. Mean ± SD, n = 3.

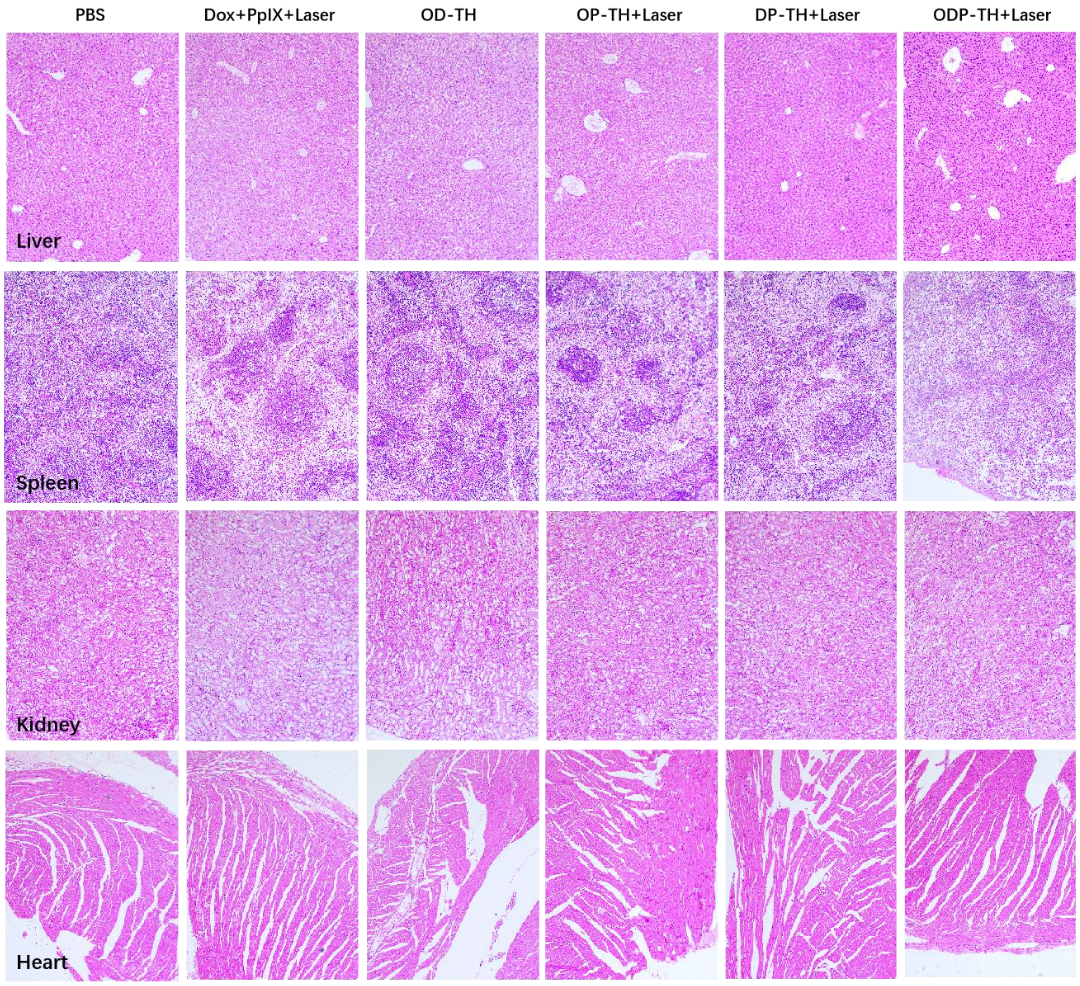

Figure S21. H&E staining images for the dissected major organs in U87MG cells bearing mice. (100x)
